# Integration of scRNA-seq data by disentangled representation learning with condition domain adaptation

**DOI:** 10.1186/s12859-024-05706-9

**Published:** 2024-03-16

**Authors:** Renjing Liu, Kun Qian, Xinwei He, Hongwei Li

**Affiliations:** School of Mathematics and Physics, China University of Geosciences (Wuhan), Wuhan, 430074 China

**Keywords:** ScRNA-seq, Cellular heterogeneity, Integration, Variational autoencoder, Disentangled representation learning, Condition domain adaptation

## Abstract

**Background:**

The integration of single-cell RNA sequencing data from multiple experimental batches and diverse biological conditions holds significant importance in the study of cellular heterogeneity.

**Results:**

To expedite the exploration of systematic disparities under various biological contexts, we propose a scRNA-seq integration method called scDisco, which involves a domain-adaptive decoupling representation learning strategy for the integration of dissimilar single-cell RNA data. It constructs a condition-specific domain-adaptive network founded on variational autoencoders. scDisco not only effectively reduces batch effects but also successfully disentangles biological effects and condition-specific effects, and further augmenting condition-specific representations through the utilization of condition-specific Domain-Specific Batch Normalization layers. This enhancement enables the identification of genes specific to particular conditions. The effectiveness and robustness of scDisco as an integration method were analyzed using both simulated and real datasets, and the results demonstrate that scDisco can yield high-quality visualizations and quantitative outcomes. Furthermore, scDisco has been validated using real datasets, affirming its proficiency in cell clustering quality, retaining batch-specific cell types and identifying condition-specific genes.

**Conclusion:**

scDisco is an effective integration method based on variational autoencoders, which improves analytical tasks of reducing batch effects, cell clustering, retaining batch-specific cell types and identifying condition-specific genes.

## Background

Single-cell RNA sequencing (scRNA-seq) has emerged as a crucial technique for quantifying gene expression at the resolution of individual cells, thus offering a powerful instrument in unraveling cell heterogeneity and deciphering the intricate molecular mechanisms that underlie diseases [1, 2]. The swift advancement of scRNA-seq technology has resulted in the accumulation of vast and diverse single-cell gene expression datasets, derived from diverse laboratories and platforms [3, 4]. These datasets encompass a wide array of species, tissue types, and experimental conditions [5]. The analysis of such heterogeneous datasets necessitates the integration of multiple scRNA-seq datasets to mitigate the influence of batch effects, which can introduce expression biases and confound biological variation [6, 7], thereby giving rise to erroneous inferences in subsequent analyses. Consequently, the correction of batch effects assumes vital importance in comprehensively investigating cellular biology. Numerous unsupervised methods for batch effect correction have been developed to overcome this challenge.

One category of methods is based on traditional approaches. These methods, inspired by the concept of Mutual Nearest Neighbors (MNN) as introduced by Haghverdi et al. [8], include Seurat v3 [9] and Scanorama [10]. In these methods, a reference dataset serves as a baseline to guide the correction of a target dataset. This correction is achieved by calculating correction vectors based on the distances between corresponding pairs of cells from the two datasets within a shared space. Another approach involves merging clustering algorithms with batch effect removal techniques. These methods cluster analogous cells from various datasets, construct joint graphs accounting for strength of connections, and subsequently apply community detection algorithms to identify cells that are intrinsically linked across different batches. Harmony [11], for instance, highlights the clustering of cells from diverse batches and associates cells from all datasets with cluster centroids. Additionally, there exist methods that rely on Non-negative Matrix Factorization (NMF), such as scINSIGHT [12], which jointly model and decompose the gene expression matrices of individual samples into condition-specific and shared modules.

Deep learning methods have also emerged as an outstanding category in the field of biology, leveraging the power of neural networks to tackle complex challenges. These approaches have attracted significant attention due to their ability to discern the underlying structure of scRNA-seq data and mitigate batch effects through intricate nonlinear transformations [13]. For instance, MMD-ResNet [14] employs a residual neural network to minimize distribution discrepancies among datasets. scVI [15] estimates parameters based on the principles of Variational AutoEncoder (VAE) [16]. DESC [17] removes batch effects through iterative clustering. Cell BLAST [18] aligns cells from different batches using a neural network-based generative model with adversarial strategies. DeepMNN [19] explores MNN pairs across batches in PCA space to minimize distances. iMAP [20] incorporates a generative adversarial network (GAN) [21] to match the distribution of shared cell types using style transfer techniques. SCIDRL [22] generates biologically meaningful and batch effect independent representations. CLEAR [23] maximizes the similarity of positive sample expression and minimizes the similarity of negative samples through a self-supervised contrastive learning framework, thereby learning shared cell expression representations across different datasets. In conclusion, deep learning-based methods effectively alleviate batch effects through their network architecture and robust fitting capabilities, outperforming traditional approaches in scalability and their capacity to handle larger and more complex scRNA-seq datasets [24].

While existing methods have demonstrated their utility in mitigating batch effects across multiple single-cell samples, they often inadvertently confound the underlying biological distinctions that exist between different conditions, such as different stages of various diseases, which can significantly impact subsequent biological analyses. scDisInFact [25] considers removing batch effects while preserving the nuanced biological signatures specific to each condition. scDisInFact, employing sophisticated modeling techniques, employs VAEs for disentanglement by optimizing seven loss functions. Moreover, to discriminate among various conditions, scDisInFact establishes separate encoders for the shared biological effects and conditional effects to facilitate distinct encoding processes from the outset and then maximizes the maximum mean discrepancy (MMD) across diverse conditions, which is used to ensure that the latent representation of each condition type is independent of both the batch and other irrelevant condition types. Different from scDisInFact and drawing inspiration from established methods in scRNA-seq data integration and cognizant of their inherent limitations, we introduce scDisco, which takes advantage of disentangled representation learning with condition domian adaptation to integrate biologically heterogeneous scRNA-seq data. scDisco, employing VAEs for disentanglement, features a reduced network complexity and superior generalization performance. Moreover, we are inclined to consider that condition-specific biology represents an integral facet of the broader biological landscape. Hence, we initially employ a shared encoder. To discriminate among various conditions, scDisco adopts a unique stance by further enhancing the disentangled condition-specific biological representations through conditional domain adaptation. This meticulous process yields finely-tuned, discriminative representations of condition-specific biology. These representations can be directly applied to identify condition-specific genes within networks, thus enhancing precision in downstream analyses. Different from other methods, scDisco transcends the mere correction of batch effects; it adeptly retains the intricate biological condition-specific information and unravels the underlying biological intricacies associated with diverse conditions. Moreover, recognizing the potential interaction among condition-specific biological effects, scDisco systematically disentangles and diminishes the confounding similarities that might exist between distinct conditions.

Specifically, scDisco takes preprocessed scRNA-seq expression matrices annotated with batch information as input, and then disentangles the shared biological representation from the condition-specific biological representation. In the process of removing batch effects, scDisco skillfully preserves the invaluable condition-specific biological information. Notably, scDisco employs domain-specific batch normalization (DSBN) [26], an ingenious technique originating from the field of domain adaptation, which plays a pivotal role in negating inter-condition similarities. We evaluated the performance of scDisco on both simulated and real datasets, comparing it with state-of-the-art methods. The experimental results demonstrate that scDisco effectively corrects for batch effects and achieves significant improvements in performance metrics compared to nine other methods. Furthermore, our experimental endeavors corroborate scDisco’s unique capacity to preserve batch-specific cell subtypes. Importantly, scDisco faithfully retains the distinct biological signatures tied to different conditions. This crucial capability facilitates the probing of condition-associated genes, exemplified by the discernment of noteworthy candidates such as NFATC3, associated with chronic obstructive pulmonary disease, and MAGEA6, linked to pancreatic ductal adenocarcinoma. These insights, discovered using scDisco, hold substantial promise for advancing the frontier of disease biology research.

## Methods

In this section, we will delve into the fundamental architecture of scDisco, detailing its implementation steps, experimental setup, and quantitative metrics. Additionally, we will introduce comparative methods and briefly outline the datasets employed in this academic study.

### An outline of the scDisco method

**Fig. 1:**
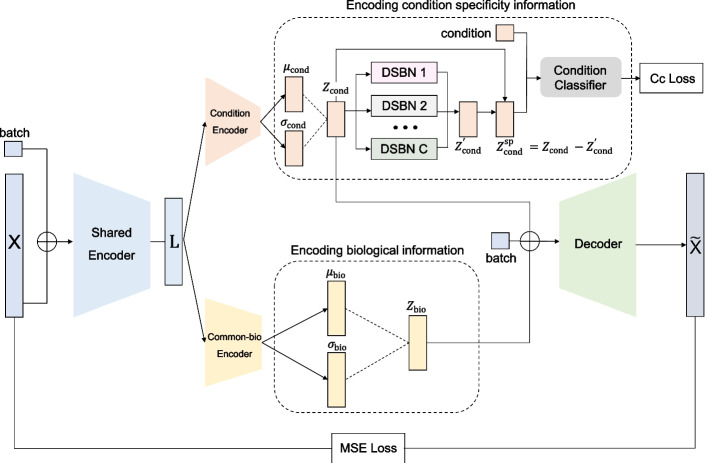
Overview of the scDisco method. scDisco comprises three key components: Shared Encoder, Encoding biological information component and Encoding condition specificity information component

The primary objective of scDisco is to minimize ambiguity between biology and technology while preserving condition-specific biological effects and extracting condition-specific genes. The method is rooted in the elegant framework of variational autoencoders, which disentangles the gene expression profiles into two independent representations: a shared biological representation and a condition-specific biological representation. scDisco comprises an array of essential components, each playing a pivotal role. These components include a shared feature encoder, a shared biological encoder, a dedicated condition encoder, condition-specific Domain-Specific Batch Normalization layers (condition-specific DSBNs), a condition classifier, and a decoder, as illustrated in Fig. 1. The encoder assumes the critical responsibility of encoding the preprocessed scRNA-seq expression matrix, annotated with batch information, into a latent representation that removes batch effects. The decoder maps the annotated latent space back to the scRNA-seq expression matrix. We introduce each of these steps in detail below.

*Shared Encoder.* scDisco takes a preprocessed scRNA-seq expression matrix, denoted as ${{\varvec{X}}} = [x_{ij}] \in {\mathbb {R}^{n \times m}}$, alongside the one-hot encoded batch information ${{\varvec{B}}} \in {\mathbb {R}^{n \times {b}}}$ as input, where $\textit{n}$ represents the total number of cells; $\textit{m}$ represents the number of genes after preprocessing; $\textit{b}$ represents the number of batches. First, the expression matrix and batch information are fed into a shared feature encoder, yielding shared features ${{\varvec{L}}} \in {\mathbb {R}^{n \times {d}}}$, which is a latent representation of dimension $\textit{d}$. Subsequently, non-shared encoders, namely the common-bio biological encoder and the condition encoder, are employed to disentangle the shared features ${{\varvec{L}}}$, resulting in two distinct representations: a shared biological representation and a condition-specific biological representation. The reconstruction process combines these representations with the batch information, ultimately generating a reconstructed matrix $\widetilde{{{\varvec{X}}}} = [\widetilde{x}_{ij}] \in {\mathbb {R}^{n \times {m}}}$. The network model is trained with a combination of reconstruction loss and the Evidence Lower Bound (ELBO) loss to remove batch effects.

*Encoding biological information.* To capture biological information, the shared features ${{\varvec{L}}}$ undergo a disentanglement process through the shared biological encoder. This operation yields the mean $\varvec{\mu }_{bio} = [({\mu }_{bio})_{ij}] \in {\mathbb {R}^{n \times {d_1}}}$ and the variance $\varvec{\sigma }_{bio}^2 = [({\sigma }_{bio}^2)_{ij}] \in {\mathbb {R}^{n \times {d_1}}}$ for the shared biological representation. Subsequently, the shared biological embedding ${{{\varvec{Z}}}}_{bio} \in {\mathbb {R}^{n \times {d_1}}}$ is obtained, with its dimension $d_1 (d_1 \ll m)$.

*Encoding condition specificity information.* Similarly, to encode condition-specific biological information, the shared features ${{\varvec{L}}}$ are disentangled using the condition encoder, yielding the mean the mean $\varvec{\mu }_{cond} = [({\mu }_{cond})_{ij}] \in {\mathbb {R}^{n \times {d_2}}}$ and the variance $\varvec{\sigma }_{\text {cond}}^2 = [({\sigma }_{\text {cond}}^2)_{ij}] \in \mathbb {R}^{n \times d_2}$ of the condition-specific biological representation. Subsequently, the condition-specific biological embedding ${\varvec{{Z}}}_{\text {cond}} \in \mathbb {R}^{n \times d_2}$ is derived, with a reduced dimension $d_2 (d_2\ll m)$. Next, ${\varvec{{Z}}}_{\text {cond}}$ serves as the input for the condition-specific DSBN layers (DSBN 1, DSBN 2, $\ldots$, DSBN C, where C represents the number of condition categories). These layers extract similarity information between conditions, resulting in the condition-shared information representation ${\varvec{{Z}}}_{\text {cond}}' \in \mathbb {R}^{n \times d_2}$ that eliminates domain-specific information. To further extract condition-specificity, the condition-specific biological embedding ${\varvec{{Z}}}_{\text {cond}}$ is differenced with the condition-shared information representation ${\varvec{{Z}}}_{\text {cond}}'$, generating the condition-specific invariant representation ${\varvec{{Z}}}_{\text {cond}}^{\text {sp}} \in \mathbb {R}^{n \times d_2}$. Finally, ${\varvec{{Z}}}_{\text {cond}}^{\text {sp}}$ is fed into the condition classifier for predicting the condition category. This aims to separate condition-specific factors from shared biological effects and prevent their removal as part of the batch effects.

Therefore, the training process of scDisco entails optimizing network weights while simultaneously minimizing reconstruction loss, ELBO loss, and condition classification loss. The outputs of scDisco comprise the shared biological embedding ${{{\varvec{Z}}}}_{cond}$ and the condition-specific invariant representation ${{{\varvec{Z}}}}_{cond}^{sp}$.

### Data pre-processing

For all scRNA-seq datasets used, scDisco employs Scanpy [27] to preprocess the raw expression matrices. The preprocessing steps are as follows: First, genes that are not expressed in fewer than one cell and cells with less than 200 expressed genes are filtered out. Second, the gene expression of each cell is normalized by its library size factor. The library size factor is defined as the total count in the cell divided by the median total count of all cells. Third, the normalized data is log-transformed, and 3000 highly variable genes are selected. These genes are then transformed into z-scores, ensuring that the expression levels of each gene have a zero mean and unit variance across all cells.

### Encoders

*Shared Encoder.* The network input involves the preprocessed single-cell gene expression profile ${{\varvec{X}}}$ accompanied by batch noise ${{\varvec{B}}}$. The extraction of shared features is facilitated through a neural network, denoted as $\text {Enc}$, which performs dimensional reduction as outlined below:

1 $$\begin{aligned} {{\varvec{L}}}=\text {Enc}({{\varvec{X}}},{{\varvec{B}}};\phi ) , \end{aligned}$$

where ${\phi }$ represents the shared layer encoder $\text {Enc}$ parameters.

Considering that condition effects are part of biological effects, while simultaneously reducing parameter counts and computational complexity, the neural network $\text {Enc}$ shares two fully connected layers. The $\text {ReLU}$ activation function is strategically employed between successive hidden layers, with the output layer also employing $\text {ReLU}$ to enforce non-negativity of outputs. Utilizing the sparsity property of the $\text {ReLU}$ function implies that each latent variable primarily activates a small subset of the data. This allows each latent feature to capture specific, unique aspects of the data and reduces computational load during training [28].

*Common-bio Encoder.* To obtain the shared biological embedding representation ${{\varvec{Z}}}_{bio}$, scDisco employs VAE that leverages the continuous and regular properties of the latent space. The VAE learns the distribution of latent variables in this space. The generation of latent variables ${{\varvec{Z}}}_{bio}$ utilizes the reparameterization technique:

2 $$\begin{aligned} & {{{\varvec{Z}}}}_{bio}=\varvec{\sigma }_{bio}^{2}\zeta _{bio} + \varvec{\mu }_{bio} , \end{aligned}$$

3 $$\begin{aligned} & \quad \varvec{\mu }_{bio}=\text {Enc}_{\varvec{\mu }_{bio}}({{\varvec{L}}};\phi _{\varvec{\mu }_{bio}})\,, \quad \varvec{\sigma }_{bio}^2=\text {Enc}_{\varvec{\sigma }_{bio}^2}({{\varvec{L}}};\phi _{\varvec{\sigma }_{bio}^2})\,, \quad \zeta _{bio}\sim \text {N}(0,{{{\varvec{I}}}}_{d_1 \times {d_1}}) , \end{aligned}$$

where $\phi _{\varvec{\mu }_{bio}}$,$\phi _{\varvec{\sigma }_{bio}^2}$ represent the parameters of the encoder networks $\text {Enc}_{\varvec{\mu }_{bio}}$ and $\text {Enc}_{\varvec{\sigma }_{bio}^2}$, respectively. Both $\text {Enc}_{\varvec{\mu }_{bio}}$ and $\text {Enc}_{\varvec{\sigma }_{bio}^2}$ are equipped with a fully connected layer that is not followed by the $\text {ReLU}$ function.

*Condition Encoder.* Similarly, the acquisition of the condition-specific biological embedding representation ${{{\varvec{Z}}}}_{cond}$ relies on leveraging the reparameterization trick:

4 $$\begin{aligned} & {{{\varvec{Z}}}}_{zond}=\varvec{\sigma }_{zond}^{2}\zeta _{zond} + \varvec{\mu }_{zond} , \end{aligned}$$

5 $$\begin{aligned} & \quad \varvec{\mu }_{zond}=\text {Enc}_{\varvec{\mu }_{zond}}({{\varvec{L}}};\phi _{\varvec{\mu }_{zond}})\,, \quad \varvec{\sigma }_{zond}^2=\text {Enc}_{\varvec{\sigma }_{zond}^2}({{\varvec{L}}};\phi _{\varvec{\sigma }_{zond}^2})\,, \quad \zeta _{zond}\sim \text {N}(0,{{{\varvec{I}}}}_{d_2 \times {d_2}}) , \end{aligned}$$

where $\phi _{\varvec{\mu }_{zond}}$,$\phi _{\varvec{\sigma }_{zond}^2}$ represent the parameters of the encoders $\text {Enc}_{\varvec{\mu }_{zond}}$ and $\text {Enc}_{\varvec{\sigma }_{zond}^2}$, respectively. $\text {Enc}_{\varvec{\mu }_{zond}}$ and $\text {Enc}_{\varvec{\sigma }_{zond}^2}$ are both equipped with a fully connected layer that is not followed by the $\text {ReLU}$ function. Further particulars can be found in section of Implementation.

### Optimizing ${{{\varvec{Z}}}}_{cond}$: attaining more specific conditional information

To enhance the specificity of the condition-specific biological embedding, scDisco employs a meticulous optimization scheme. By refining the encoding process, scDisco aims to capture intricate and comprehensive information related to the specific experimental conditions.

*Condition-specific DSBNs.* In the field of domain adaptation in deep learning, Batch Normalization is a widely used technique for normalization that expedites the convergence of neural networks. Drawing inspiration from domain adaptation, our method aims to differentiate domain-specific information from domain-invariant information, thereby improving generalization performance. To achieve this, we introduce condition-specific Domain-Specific Batch Normalization layers (DSBNs), which optimize the condition-specific biological representations ${{{\varvec{Z}}}}_{{cond}_{c}}$. The DSBNs incorporate multiple branches of Batch Normalization (BN) layers, customized to the number of condition types. Each branch is assigned to a particular conditional domain.

By retaining BN for each condition domain and independently normalizing data from different condition sources, DSBNs assign domain-specific learnable scaling and shifting parameters for each conditional domain label $c \in 1,2,$ $\ldots$ $,C$:

6 $$\begin{aligned} {{{\varvec{Z}}}}_{{cond}_{c}}^{'}[i,j] = \frac{{{{\varvec{Z}}}}_{{cond}_{c}}[i,j]-\mu _c}{\sqrt{\sigma _{c}^2+\epsilon }} , \end{aligned}$$

where $\epsilon$ is a small constant used to prevent division by zero. Therefore, ${{{\varvec{Z}}}}_{{cond}_{c}}^{'}$ represents the similarity across conditions. It’s extracted by removing the mean and variance specific to different conditions, effectively eliminating the differences caused by varying conditions.

After being processed by the condition-specific DSBNs, the representation of conditional shared information ${{{\varvec{Z}}}}_{{cond}_{c}}^{'}$ effectively reduces the condition-specificity among different types of conditions. By applying a subtraction operation to each conditional field, we obtain more condition-specific representations to each condition domain:

7 $$\begin{aligned} {{{\varvec{Z}}}}_{{cond}_{c}}^{sp} = {{{\varvec{Z}}}}_{{cond}_{c}} - {{{\varvec{Z}}}}_{{cond}_{c}}^{'} {.} \end{aligned}$$

This step aims to retain condition-specific information while removing differences in mean and variance caused by different conditions.

*Classifier.* In order to maximize the discrimination of conditional heterogeneity factors and protect them from being mistaken for batch-related noise, scDisco introduces a conditional classifier denoted as $\text {Cls}$. This classifier is constructed utilizing a neural network endowed with learnable parameters represented by $\phi _{\text {Cls}}$. scDisco proceeds by inputting the conditioned latent variable ${{{\varvec{Z}}}}_{{cond}_{c}}^{sp}$ into the conditional classifier, which contains richer and more specific information about the conditions. $\text {Cls}$ serves the purpose of condition label classification, producing probabilities for each cell’s membership within various classes specific to the given condition. The dissimilarity between the predicted conditional probability distribution and the true conditional distribution is quantified through the utilization of the cross-entropy loss function:

8 $$\begin{aligned} loss_{\text {Cls}} = -c_t\text {log}\text {Cls}({{{\varvec{Z}}}}_{{cond}_{c}}^{sp};\phi _{\text {Cls}}) , \end{aligned}$$

where $c_t$ represents the true condition label, encoded in a one-hot representation; the neural network $\text {Cls}$ employs fully connected layers, incorporating $\text {ReLU}$ activation functions within its intermediate layers. Configuration details can be found in section of Implementation.

### Decoder and loss function

We model ${{\varvec{X}}}$ as a joint generation of embedding variables ${{{\varvec{Z}}}}_{bio}$ within a $d_1$-dimensional shared biological latent space, ${{{\varvec{Z}}}}_{cond}$ within a $d_2$-dimensional condition-specific biological latent space, and batch noise ${{\varvec{B}}}$. The decoding procedure in scDisco employs a fully connected network called $\text {Dec}$, parameterized by $\theta$. $\text {Dec}$ serves the purpose of mapping both the biological latent space and the condition-specific biological latent space back to the preprocessed gene expression space, as expressed by the equation:

9 $$\begin{aligned} \widetilde{{{\varvec{X}}}} = \text {Dec}({{\varvec{Z}}}_{bio},{{\varvec{Z}}}_{cond}, {{\varvec{B}}};\theta ) , \end{aligned}$$

where Dec comprises three fully connected layers. Details can be found in section of Implementation.

While the derivation of the posterior probability distribution $p{({{{\varvec{Z}}}}|{{{\varvec{X}}}},{{{\varvec{B}}}})}$ involves intricate computations related to the marginal distribution $p({{{\varvec{X}}}}|{{{\varvec{B}}}})$, necessitating integration over latent variables, we introduce a variational distribution $q_\phi ({{{\varvec{Z}}}}| {{{\varvec{X}}}},{{{\varvec{B}}}})$. This distribution is defined by an encoder neural network, aiming to provide an approximate of the posterior distribution $p({{{\varvec{Z}}}}|{{{\varvec{X}}}},{{{\varvec{B}}}})$, where $\phi$ represents the learnable parameters of the encoder. In the subsequent sections, we present the pivotal steps of this process, with comprehensive derivations available in the Supplementary Methods of Additional file 1 for further reference.

Utilizing Monte Carlo estimation and employing the Kullback–Leibler (KL) divergence, we measure the similarity between $p({{{\varvec{Z}}}}| {{{\varvec{X}}}},{{{\varvec{B}}}})$ and $q_\phi ({{{\varvec{Z}}}}| {{{\varvec{X}}}},{{{\varvec{B}}}})$ with the objective of minimizing the KL divergence $\mathrm {D_{KL}}(q_\phi ({{{\varvec{Z}}}}| {{{\varvec{X}}}},{{{\varvec{B}}}})\ ||\ p({{{\varvec{Z}}}}|{{{\varvec{X}}}},{{{\varvec{B}}}}))$. This endeavor gives rise to the following optimization problem:

10 $$\begin{aligned} \min _{\phi }{\mathrm {D_{KL}}}\left( q_\phi ({{{\varvec{Z}}}}| {{{\varvec{X}}}},{{{\varvec{B}}}})\ ||\ p({{{\varvec{Z}}}}|{{{\varvec{X}}}},{{{\varvec{B}}}})\right) . \end{aligned}$$

The act of minimizing the KL divergence as described in Eq. (10) is equivalent to the maximization of the evidence lower bound (ELBO) [29], expressed as follows:

11 $$\begin{aligned}&\min _{\phi }{\mathrm {D_{KL}}}\left( q_\phi ({{{\varvec{Z}}}}| {{{\varvec{X}}}},{{{\varvec{B}}}})\ ||\ p({{{\varvec{Z}}}}|{{{\varvec{X}}}},{{{\varvec{B}}}})\right) \\&\quad = \max _{\phi ,\theta }{\textrm{ELBO}}\\&\quad = \max _{\phi ,\theta } \Big \{ {\mathbb {E}_{z\sim q}[\log {p_{\theta }({{{\varvec{X}}}}| {{{\varvec{Z}}}},{{{\varvec{B}}}})}] - {\mathrm {D_{KL}}}\left( q_\phi ({{{\varvec{Z}}}}| {{{\varvec{X}}}},{{{\varvec{B}}}})\ ||\ p({{{\varvec{Z}}}})\right) } \Big \} . \end{aligned}$$

In Eq. (11), the distribution $p_{\theta }({{{\varvec{X}}}}| {{{\varvec{Z}}}},{{{\varvec{B}}}})$ follows a Gaussian distribution $\textrm{N}\left( \textrm{Dec}\left( {{{\varvec{Z}}}},{{{\varvec{B}}}}\right) ,c{{{\varvec{I}}}}\right)$. Assuming the separability of the biological latent space and the condition-specific biological latent space, our Multi-facet variational distribution $q_\phi ({{{\varvec{Z}}}}| {{{\varvec{X}}}},{{{\varvec{B}}}})$ [30] adopts a mean-field form:

12 $$\begin{aligned} q_\phi ({{{\varvec{Z}}}}| {{{\varvec{X}}}},{{{\varvec{B}}}}) = q_\phi ({{{\varvec{X}}}},{{{\varvec{B}}}})\prod _{j=1}^{J}q_\phi ({{{\varvec{Z}}}}_j| {{{\varvec{X}}}},{{{\varvec{B}}}}) , \end{aligned}$$

where *J*=2. For each *j*, the variational distribution $q_\phi ({{{\varvec{Z}}}}_j| {{{\varvec{X}}}},{{{\varvec{B}}}})$ takes the form of a multivariate Gaussian distribution featuring a diagonal covariance matrix, and its mean and variance are determined by the encoder $\text {Enc}_{\varvec{\mu }_{bio}}$, $\text {Enc}_{\varvec{\mu }_{cond}}$, $\text {Enc}_{\varvec{\sigma }_{bio}^2}$ and $\text {Enc}_{\varvec{\sigma }_{cond}^2}$.

As a result, our VAE loss function can be defined as follows:

13 $$\begin{aligned} \begin{aligned} loss_{\text {VAE}}&= -\textrm{ELBO} \\&= \mathrm {D_{KL}}\left( q_\phi ({{{\varvec{Z}}}}| {{{\varvec{X}}}},{{{\varvec{B}}}})\ ||\ p({{{\varvec{Z}}}})\right) - \mathbb {E}_{z\sim q}[\log {p_{\theta }({{{\varvec{Z}}}}| {{{\varvec{X}}}},{{{\varvec{B}}}})}] \\&= \sum _{j=1}^{J}[\mathrm {D_{KL}}\left( q_\phi ({{{\varvec{Z}}}}_j| {{{\varvec{X}}}},{{{\varvec{B}}}})\ ||\ p({{{\varvec{Z}}}}_j)\right) ] - \mathbb {E}_{z\sim q}[\log {p_{\theta }({{{\varvec{Z}}}}| {{{\varvec{X}}}},{{{\varvec{B}}}})}] \\&= \sum _{i=1}^{m}\sum _{j=1}^{n}||x_{ij}-{\widetilde{x}}_{ij}||^2 \\&\quad + \lambda \Big (\frac{1}{2}\sum _{i=1}^{d_1}\sum _{j=1}^{n}((\mu _{bio})_{ij}^2 + (\sigma _{bio})_{ij}^2 - 1 - \textrm{log}(\sigma _{bio})_{ij}^2) \\&\quad + \frac{1}{2}\sum _{i=1}^{d_2}\sum _{j=1}^{n}((\mu _{cond})_{ij}^2 + (\sigma _{cond})_{ij}^2 - 1 - \textrm{log}(\sigma _{cond})_{ij}^2)\Big ) \\&\overset{\Delta }{=}\ loss_1 + \lambda \ {loss_2} . \end{aligned} \end{aligned}$$

The objective function of scDisco can be formulated as follows:

14 $$\begin{aligned} loss = loss_1 + \lambda \ loss_2 + \mu \ loss_{\text {Cls}} , \end{aligned}$$

where the first term in the objective function aims to minimize the reconstruction error; the second term represents the $\text {ELBO}$ loss; the third term corresponds to the conditional classification loss; $\lambda$ and $\mu$ are coefficients used to weigh the importance of these losses, respectively.

### Condition-specific genes selection

For each dataset, assuming we have C sets of conditions, we sequentially use each condition (e.g., condition $c_1$) to treat the cells in that set as the query dataset, while considering the remaining conditions (e.g., $c_2,c_3,$ $\ldots$ $,c_C$) as the reference dataset. Within this framework, we calculate the condition-specific domain-invariant representation deviations ($\Delta {{\varvec{z}}}_{cond}^{sp} \in {\mathbb {R}^{d_2}}$), which quantify the distinctions between the query cells and the reference cells. Subsequently, we perform backpropagation of gradients through the neural network, directing them back to the input gene expression space:

15 $$\begin{aligned} \Delta {\widetilde{{{\varvec{x}}}}} = \left( \frac{\partial {{{\varvec{z}}}_{cond}^{sp}}}{\partial {\widetilde{{{\varvec{x}}}}}}\right) ^{\intercal }\cdot \Delta {{{\varvec{z}}}}_{cond}^{sp} , \end{aligned}$$

where $$\frac{\partial {{{\varvec{z}}}_{cond}^{sp}}}{\partial {\widetilde{{{\varvec{x}}}}}} \in \mathbb {R}^{d_2 \times m}$$ and $$\Delta {\widetilde{{{\varvec{x}}}}} \in \mathbb {R}^{m}$$.

To provide greater precision, scDisco initiates by employing a stochastic process. Specifically, we randomly select five cells from the reference dataset for each cell within the query dataset, thereby strengthening the robustness of our model. Subsequently, we compute the average of the embedded representations for these five cells and normalize the deviation relative to the query cell, yielding a unit vector denoted as $\Delta {{\varvec{z}}}_{cond}^{sp}$. This unit vector provides us with the gene gradients that represent the movement of cells from other conditions towards the query condition.

Following this, we engage in backpropagation, utilizing the gradients of the embedded representation pertaining to the query cell, with the normalized deviations serving as gradient values. This procedure provides us with gene gradient values for all cells operating under that specific condition.

Consequently, we proceed to determine the average gene gradient value $\Delta {\widetilde{{{\varvec{x}}}}}$ for each gene within this condition. Increased gradient values serve as indicators of heightened gene expression levels, thereby contributing significantly to the directional movement of cell embeddings towards the specific condition in focus. This process effectively identifies these genes as pivotal features within that condition.

In conclusion, we conduct a sorting operation on all gene gradient values associated with each condition, subsequently selecting the top 15 genes distinguished as the most significant contributors.

### Implementation

scDisco is implemented in Python 3.8, using Scanpy version 1.9.3 for data preprocessing. Within our model architecture, the shared feature encoder and decoder are equipped with hidden layers, their dimensions thoughtfully configured as 512, 256, dim, 256, 512, with dim representing the dimensionality of the bottleneck layer. The dimension of the common-bio encoder is determined based on the number of cells, and one can find detailed specifications in Table S1 of Supplementary Tables [see Additional file 1]. Concurrently, the dimension of the condition encoder is set to 8. We use the Adam for optimization with a learning rate of 0.0001 and train scDisco for a total of 50 epochs for real datasets. The hidden layer sizes of the condition classifier are set as 100, 100, 100, 100, and the Adam optimizer is used with a learning rate of 0.0001. The loss function weights are set as hyperparameters with $\lambda =0.001$ and $\mu =0.001$. The analysis of how to choose hyperparameters $\lambda =0.001$ and $\mu =0.001$ can be found in the Table S2–S9 of Supplementary Tables [see Additional file 1].

For thorough quantitative evaluation across all datasets, we employ a suite of robust metrics. These include ARI [31] and NMI [32], which provide insights into clustering quality. ARI compares known cell type labels with the integrated clustering results, ranging from −1 to 1, where a value closer to 1 indicates better clustering performance. Comparing known cell type labels with the integrated clustering results, the NMI ranges from 0 to 1, where a value closer to 1 indicates better clustering performance. Additionally, we utilize two integrated metrics ASW [33] and F1 Score [7], which both represent a combination of batch-wise ASW (BASW) and cell-wise ASW (CASW). ASW ranges from 0 to 1, with a higher ASW indicating better batch correction effectiveness. And a higher F1 Score indicates better batch correction effectiveness. The clustering labels are efficiently computed using the k-means algorithm. We use ${{{\varvec{Z}}}}_{bio}$ for UMAP visualization and the above metrics calculations, and we employ ${{\varvec{Z}}}_{cond}^{sp}$ for condition UMAP visualization and identification of associated genes. Comprehensive details pertaining to these evaluation metrics, a more detailed explanation of the rationale behind our data selection and the data selection of comparison methods for plotting and clustering can be referenced in the Additional file 1: Sect. 3.1.

For datasets containing fewer than 10,000 cells, we adopt a meticulous approach to mitigate algorithmic variability and ensure fair comparisons. scDisco entails computing the above metrics by performing ten iterations of sampling, each involving 95$\%$ subsampling per class of cell from the original expression data. When conducting ten repetitions of the sampling experiment with a dataset size exceeding 10,000 cells, certain methods lead to an extension of the computational runtime to more than 48 h. Therefore, for datasets surpassing the 10,000-cell threshold, considering memory consumption and resource limitations, we perform experiments on the complete dataset without sampling for comparison. In particular, for datasets with more than 20,000 cells when calculating F1 Score and ASW, we do not perform 20 repeated samplings to evaluate certain comparative methods due to their time and memory requirements. Instead, we only calculate the results once without sampling, while still conducting sampling in all other experiments.

### Integration methods used for comparison

To assess the efficacy of the scDisco method, we compare it with nine other methods: Seurat [9], Harmony [11], Scanorama [10], DESC [17], scVI [15], Cell BLAST [18], SCIDRL [22], scDisInFact [25], and scINSIGHT [12]. Specifically, Seurat, Harmony, and Scanorama are classical traditional methods that do not utilize neural networks. DESC employs a self-encoding network framework for batch effect removal through the concept of deep embedded clustering, whereas our method operates within an enhanced variational autoencoder framework. Additionally, we contrast our approach with scVI and Cell BLAST, both of which employ a variational autoencoder framework. SCIDRL is a method based on the resolution of confounding by separating batch noise, while our method utilizes the idea of disentangling to separate condition-specific biological effects. Furthermore, we compare our method with scDisInFact and scINSIGHT, methods that address heterogeneous samples originating from different biological conditions and separate these condition biological effects. To ensure the fairness of the comparison experimental results, we have not made any modifications to the code and tutorials provided by the authors, excluding the mismatch between the tutorials and the original paper descriptions. All comparison methods are implemented using the default parameters and the default preprocessing steps.[see Additional file 1: Tables S10 and S11].

The experiments were carried out on a workstation with an Intel(R) Iris(R) Xe graphics card, eight 11th Gen Intel(R) Core(TM) i5-1155G7 @ 2.50GHz CPUs, and 16 G random access memory (RAM). It’s worth noting that we performed all experiments for all methods in the CPU environment.

### Datasets

The simulated dataset used in our study was generated using the scDesign R package [34]. It consists of 3000 cells and 4977 genes. This dataset was designed to simulate data from three time points (T1, T2, T3), with two samples at each time point, resulting in a total of six distinct cell types (c1, c2, c3, c4, c5, c6). Notably, the cell types c1, c2, and c3 are present across all samples, while the cell types c4, c5, and c6 are only found under specific conditions (Table 1).

For the real datasets, we acquired six publicly available datasets with cell annotation labels. These datasets encompass five human datasets and one mouse dataset. Comprehensive details and download links for these six datasets are provided in the Supplementary Methods of Additional file 1, and the specific quantity information is summarized briefly in Table 2.

**Table 1:** Cell type compositions in the simulated dataset

Condition	Sample	Cell type					
		c1	c2	c3	c4	c5	c6
T1	S1	100	100	100	0	100	100
	S2	100	100	100	0	100	100
T2	S3	100	100	100	100	0	100
	S4	100	100	100	100	0	100
T3	S5	100	100	100	100	100	0
	S6	100	100	100	100	100	0

**Table 2:** Quantitative information of the six real datasets

Datasets	No. of cells	No. of genes	No. of groups	No. of batches
Human pancreas2 [35]	2914	30415	13	10
Human lung [36]	3566	19206	14	4
Human pancreas [37]	8569	20125	14	4
Mouse mucosa [38]	43677	30446	24	4
Human epithelium [39]	32926	26528	10	15
Human ductal [40, 41]	57530	18008	10	35

## Results

To evaluate the performance of scDisco in the integration analysis of scRNA-seq data, we conducted experiments on both simulated and real datasets. By comparing it with nine other integration methods, we demonstrated that scDisco possesses an advantage in batch effect removal and can extract condition-related significant genes. All comparative methods were carefully executed in strict adherence to their respective recommended standard pipelines.

### scDisco improves cell-type resolution and batch-effect correction on simulated data

In order to benchmark the performance of the scDisco method, we conducted experiments using simulated scRNA-seq data with known cell type composition and condition-specific effects as outlined in Table 1.

**Fig. 2:**
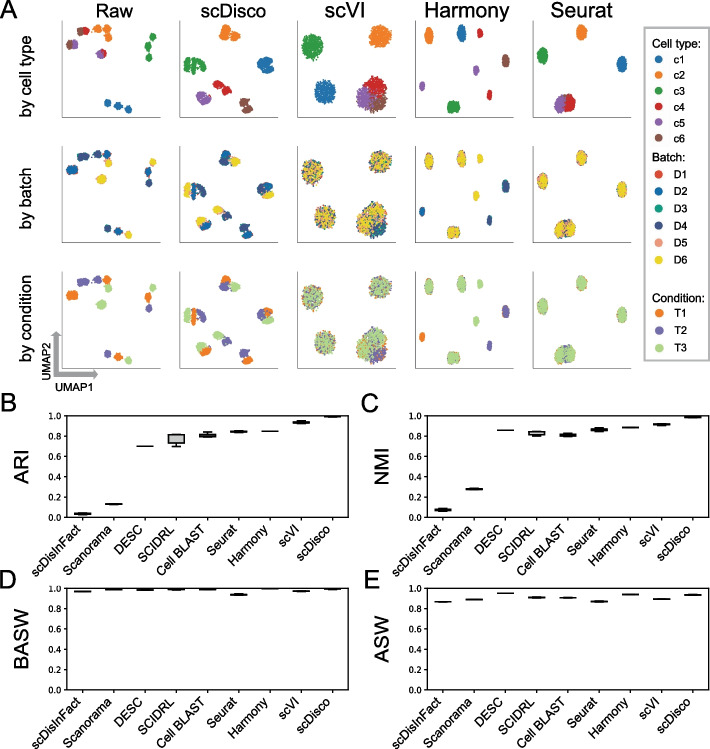
The integrated comparation of the simulated dataset. **A** UMAP plots of Raw and the cell embeddings produced by scDisco, scVI, Harmony, and Seurat. Each point represents a cell, and each column represents a method, while each row corresponds to the UMAP plot with coloring based on true cell types, batch IDs, and condition IDs. **B–E** Boxplots of ARI, NMI, BASW, and ASW values of each method by applying the nine integration methods to randomly selected subsamples of the complete simulated data

For raw data, we observed that the data exhibited a phenomenon where the true cell types were indistinguishable due to variations in cell types, batches, and conditions, as illustrated in Fig. 2A. And cells, for which batch effects are not initially removed, are influenced by specific time points, leading to clustering patterns that may be misleading. Taking into account the influence of random factors, we performed ten repeated experiments for each method on the simulated dataset, with comprehensive details on the experimental settings available in the Implementation section. When running ten sampling iterations on simulated data with scINSIGHT, the computation time will be extended to over 10 h. Therefore, we applied only scDisco alongside eight other comparative methods—Seurat, Harmony, Scanorama, DESC, scVI, Cell BLAST, SCIDRL, scDisInFact to the simulated dataset, using time points as the condition factor. Among these methods, we visualized the UMAP plots based on the average ARI values calculated from ten runs for each method. These plots were organized in descending order to facilitate comparison. Figure 2 showcases the visualizations for four of these methods, while additional visualizations for the remaining methods can be found in Additional file 1: Fig. S1.

The visual representations clearly demonstrate the successful identification and segregation of the true cell types accomplished by scDisco (Fig. 2A). In addition, our method effectively retains specific batch-related effects, as evidenced by the UMAP plots wherein cells c4, c5, and c6 remain adequately preserved and separate, despite their presence in distinct batches. Conversely, the other methods fail to segregate cells c4, c5, and c6 (Figs. 2A, S1A). Using the known ground truth cell labels, we computed the NMI and ARI scores, which indicate that scDisco achieves results close to 1 and exhibits remarkable stability (Fig. 2B, C), in line with the ground truth. Additionally, scDisco attains high scores in the computation of BASW and ASW metrics (Fig. 2D, E), indicating that the distances between cells from different batches are similar to within-batch distances, while the distances between cells of the same cell type are also relatively close. To visually present the distances between different cell clusters on the plot, we utilized PCA for linear dimensionality reduction and visual analysis, as shown in Additional file 1: Fig. S2. And we quantitatively assessed whether cells of the same type after integration became dissimilar, which can be found in Additional file 1: Table S13.

### scDisco identifies batch-specific cell types

**Fig. 3:**
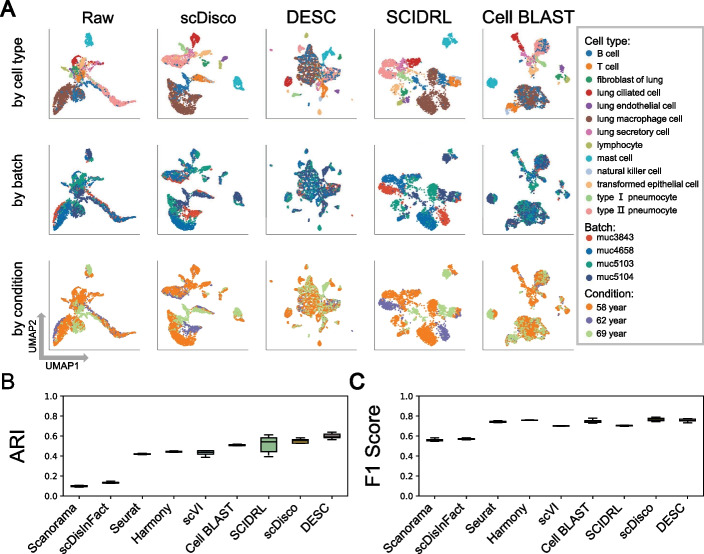
The integrated comparation of the human lung dataset. **A** UMAP plots of Raw and the cell embeddings produced by scDisco, DESC, SCIDRL, and Cell BLAST. Each point represents a cell, and each column represents a method, while each row corresponds to the UMAP plot with coloring based on true cell types, batch IDs, and condition IDs. **B–C** Boxplots of ARI and F1 Score values of each method by applying the nine integration methods to randomly selected subsamples of the complete human lung data

To further validate the ability of scDisco in preserving batch-specific cell types, we performed experiments on the dataset of human lung. For the human lung, we first considered data cleaning by removing cells with unknown true cell types and eliminating cell types with a count below one percent. As a result, the dataset comprised 3202 cells belonging to 13 distinct cell types across 4 batches. Among these batches, Muc3843, Muc4658, and Muc5103 batches shared nine cell types with the Muc5104 batch. Notably, the Muc5104 batch exhibited unique cell types, including lung ciliated cells, transformed epithelial cells, lung secretory cells, and type II pneumocytes cells. The eight comparative methods were ranked based on the average ARI values calculated from ten iterations. The UMAP plots were visualized in a descending order, and here we only present the visualizations of three comparative methods in Fig. 3A. The remaining methods can be found in Additional file 1: Fig. S3.

scDisco effectively separated lung ciliated cells and transformed epithelial cells, with minimal admixture of lung secretory cells with other cell types. Type II pneumocytes cells were mainly separated as well, highlighting the ability of scDisco to preserve batch-specific cell types (Fig. 3A). DESC managed to separate a significant portion of lung ciliated cells, but it exhibited a slight mixture with B cells, and type II pneumocytes cells could not be distinguished from B cells (Fig. 3A). SCIDRL divided type II pneumocytes cells into two clusters and failed to cluster transformed epithelial cells.

Although both Cell BLAST and scVI successfully separated lung ciliated cells, they exhibited some degree of mixing with lung secretory cells and type II pneumocytes cells, along with other cell types (Fig. 3A). Harmony failed to separate lung secretory cells and mixed type II pneumocytes cells with B cells. Seurat failed to separate transformed epithelial cells, lung secretory cells, and type II pneumocytes cells. scDisInFact failed to distinguish type II pneumocytes cells from B cells. Scanorama was unable to separate the four batch-specific cell types belonging to Muc5104 [see Supplementary Figures of Additional file 1, Fig. S3A].

Additionally, except for scDisco, which showed minimal mixing between lung macrophage cells with B cells, all other methods resulted in a significant mixture between these two cell types. Moreover, apart from scDisco and SCIDRL, other methods showed considerable mixing between type II pneumocytes and B cells. In terms of performance metrics, scDisco achieved higher ARI scores and the highest F1 score values (Fig. 3B, C). Additionally, it also exhibited superior values in NMI and ASW scores [see Supplementary Figures of Additional file 1: Fig. S3B, C].

To evaluate the performance of scDisco on integrating datasets with a limited number of shared common cell types while preserving batch-specific cell type clusters, we constructed a specialized dataset with only one shared cell type, named Human pancreas-subset [see Detailed description of the real datasets]. Then, we conducted integration using scDisco and nine comparison methods (Additional file 1: Fig. S4 of Supplementary Figures). scDisco could effectively retain and separate all nine cell types, with only a slight mixture between endothelial cells and acinar cells, as shown in Additional file 1: Fig. S4A. And scDisco achieved the highest ARI, demonstrating favorable outcomes in batch mixing and preserving cell purity, as observed in Additional file 1: Fig. S4B, C.

### scDisco achieves effective integration of multiple batches on datasets with varying species and data sizes

To evaluate the performance of scDisco on datasets with varying numbers of batches, species, and data sizes, we conducted experiments using simulated data and five datasets: a small-scale dataset called human pancreas2 with 10 batches, a small-scale dataset called human lung with 4 batches, a slightly larger human pancreas dataset with 4 batches, a mouse mucosa dataset with 4 batches, and a large-scale human epithelium dataset with 15 batches. Due to the significant time and memory requirements of scINSIGHT, especially when confronted with datasets of a scale surpassing 20,000 cells, scINSIGHT cannot generate results, even after exceeding a runtime of 96 h. Consequently, our experimental purview was limited to encompass solely the simulated data and four real datasets: human pancreas2, human lung, human pancreas, and mouse mucosa. For other methods, we have shown results of simulated data and results of human lung in the preceding two sections. Before the analysis, we conducted data cleaning for all real datasets, removing cells with unknown cell types and cells from types that represented less than 1$\%$ of the total cell count. As a result, the human pancreas2 dataset was reduced to 2,125 cells with 8 cell types across 10 batches, the human pancreas dataset was reduced to 8,451 cells with 9 cell types across 4 batches, the mouse mucosa dataset was reduced to 10,974 cells with 17 cell types across 4 batches, and the human epithelium dataset was reduced to 24,163 cells with 5 cell types across 15 batches.

**Fig. 4:**
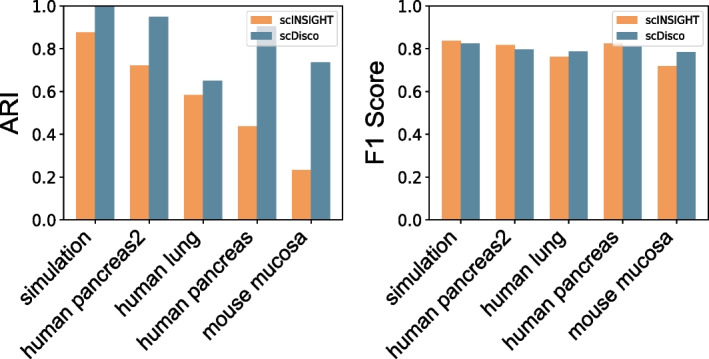
Bars of ARI and F1 Score of the five datasets by scINSIGHT and scDisco

The scINSIGHT model was trained using the default parameters. Additionally, no sampling was performed on any of the five datasets. Specifically, we trained scINSIGHT and scDisco models using the five datasets individually and generated UMAP plots [see Supplementary Figures of Additional file 1: Fig. S9] along with the evaluation metrics ARI and F1 Score (Fig. 4). The results revealed that the batch effect removal effectiveness of the scDisco model on the UMAP plots was generally superior, with enhanced separation between cell clusters. In the simulated data, the scINSIGHT model struggled to completely distinguish between the two cell types [see Supplementary Figures of Additional file 1: Fig. S9A]. However, for the other four real datasets, scDisco consistently achieved enhanced separation between cell clusters, as well as more cohesive clustering within clusters. Upon analyzing the integrated UMAP plots, it became apparent that the mixing performance of scDisco was comparable to that of scINSIGHT. Across all five datasets, scDisco yielded higher ARI scores (Fig. 4). Except for the simulated data and human pancreas2, scINSIGHT demonstrated higher F1 Score results, whereas scDisco had superior F1 Score performance on the remaining datasets.

For the comparison methods other than scINSIGHT, we conducted experiments separately on human pancreas2, human pancreas, mouse mucosa, and human epithelium and compared them with scDisco. For the human pancreas2 dataset, scDisco demonstrated effective segregation for most cell types, although there was some minor co-expression cell mixing with type B pancreatic cells [see Supplementary Figures of Additional file 1: Fig. S5A]. In the case of the human pancreas dataset, scDisco successfully achieved segregation for the 9 cell types, although ductal cells exhibited some dispersion, and a small number of beta cells were intermingled with delta cells [see Supplementary Figures of Additional file 1: Fig. S6A]. For the mouse mucosa dataset, scDisco achieved basic separation for the 17 cell types and exhibited relatively tight clustering, with only a few cell types split into two clusters [see Supplementary Figures of Additional file 1: Fig. S7A]. Furthermore, scDisco obtained the highest ARI, NMI, and F1 Score values [see Supplementary Figures of Additional file 1: Fig. S7B–E]. Regarding the human epithelium dataset, scDisco, scVI, and cell BLAST showed slightly lower separation compared to DESC for different cell types, with some mixing of KRT8-expressing intermediate cells with respiratory basal cells. However, they were still able to obtain reasonably tight cell clusters [see Supplementary Figures of Additional file 1: Fig. S8A].

### scDisco achieves effective integration of multiple batches on large-scaled data

**Fig. 5:**
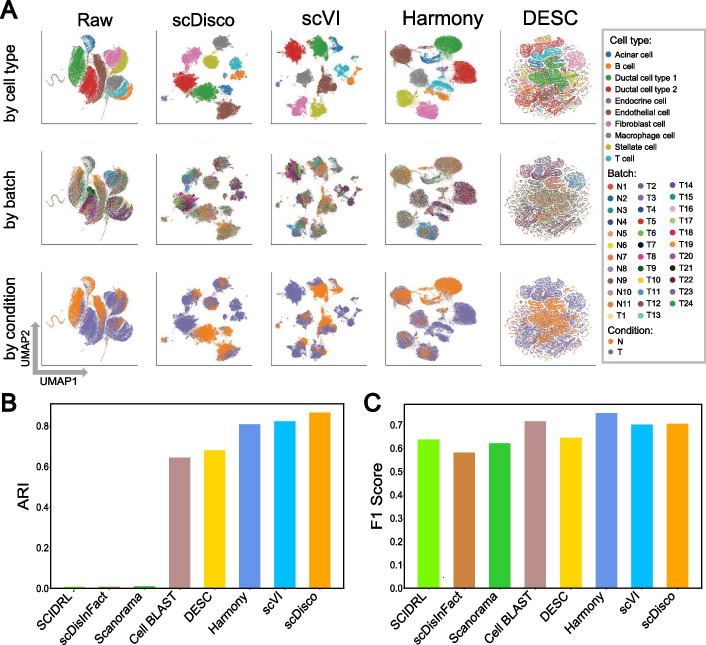
The integrated comparation of the human ductal dataset. **A** UMAP plots of Raw and the cell embeddings produced by scDisco, scVI, Harmony and DESC. Each point represents a cell, and each column represents a method, while each row corresponds to the UMAP plot with coloring based on true cell types, batch IDs, and condition IDs. **B–C** Bars of ARI and F1 Score of the eight integration methods of the complete human ductal data

Furthermore, to evaluate the performance of scDisco on datasets characterized by larger sizes and a greater number of batches, we conducted experiments using the human ductal dataset and compared it against seven alternative methods, excluding Seurat and scINSIGHT. Seurat was not included in the comparative analysis due to its substantial resource requirements surpassing the memory capacity of our device, and the same applies to scINSIGHT. The seven methods were ranked according to the calculated ARI, and the UMAP visualizations were plotted in descending order. Figure 5 only displayed the visualizations for three of the comparative methods, while the visualizations for the remaining methods are presented in Additional file 1: Fig. S10. Notably, among the 11 batches (N1 to N11), none of them contained ductal cell type 2. And none of the 5 batches (T1, T3, T14, T18, T22) included ductal cell type 2.

scDisco has successfully achieved separation and clustering of cell populations, effectively distinguishing ductal cell type 1 from ductal cell type 2 (Fig. 5A). scVI exhibited slight mixing of a small portion of ductal cell type 1 with ductal cell type 2, along with some fusion between ductal cell type 2 and acinar cells. Harmony mixed of a small portion of ductal cell type 1 with ductal cell type 2 and mixed T cells with other cells. DESC showed scattered results, with several cell clusters mixing with each other, and there was no distinct separation observed. Regrettably, Cell BLAST was unable to effectively differentiate ductal cell type 1 from ductal cell type 2. Scanorama, scDisInFact, and SCIDRL mostly mixed all cells together [see Additional file 1: Fig. S10]. Experimental results have confirmed that scDisco has not only conserved batch-specific cells but also accomplished commendable clustering outcomes. In terms of performance metrics, scDisco has garnered the highest ARI, as depicted in Fig. 5B.

Concurrently, we transformed the variational autoencoder network into an autoencoder network to verify the effectiveness of the variational autoencoder. Specifically, we employed six real datasets to train the scDisco-VAE model and the scDisco-AE model. The results demonstrated that the scDisco-VAE model yielded superior batch effect removal effects in the UMAP plots, with enhanced separation between cell clusters and a more cohesive clustering within clusters [see Supplementary Figures of Additional file 1: Fig. S12A]. Moreover, when evaluating the ARI metric across the six real datasets, the results consistently favored the scDisco-VAE model as illustrated in Additional file 1: Fig. S12B. Notably, except for the human epithelium dataset, the F1 Score metric also revealed higher values for the scDisco-VAE model.

### scDisco identifies condition-specific genes

**Fig. 6:**
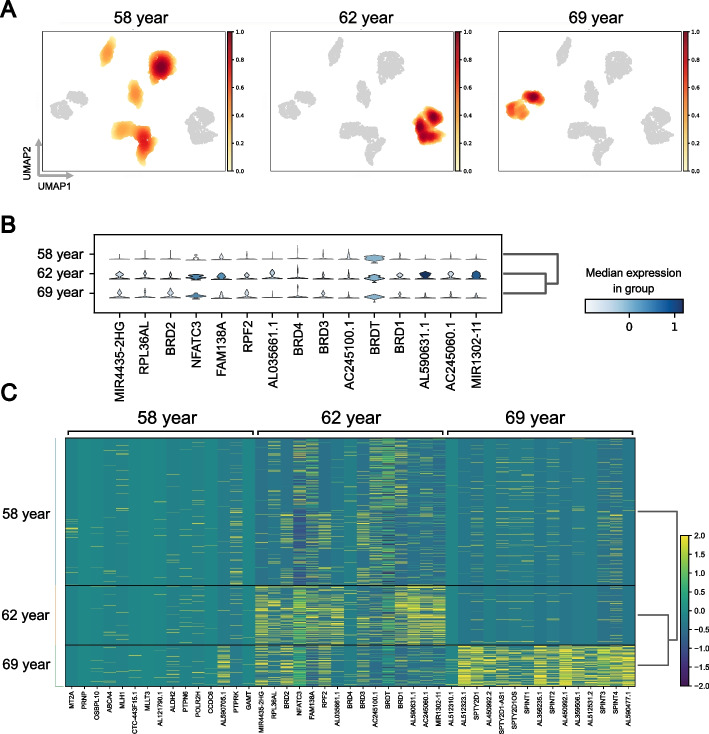
Condition-specific genes of the human lung. **A** The UMAP density plot of the condition-specific invariant embedding based on age: 58 year, 62 year, and 69 year. **B** The violin plot of the gene expression distribution specifically for the 62-year-old condition. The width of the violin indicates the concentration of gene expression values, while its height represents the range of gene expression values. **C** The heatmap of the gene expression matrix displays the condition-specific gene expression patterns for the three categories: 58 years, 62 years, and 69 years. The deeper the shade of yellow, the higher the gene expression value

**Fig. 7:**
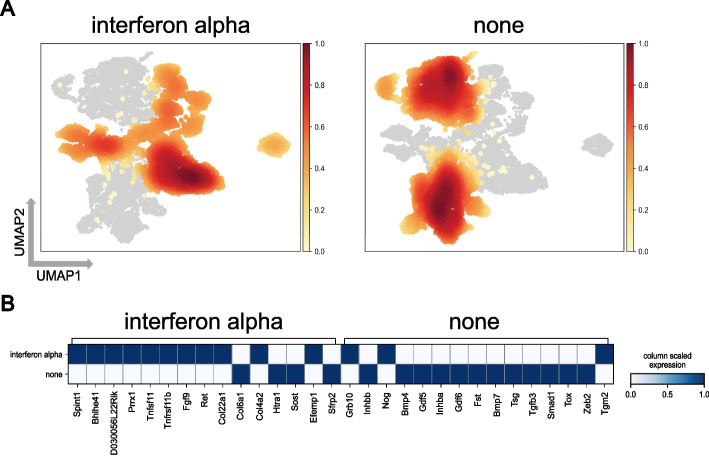
Condition-specific genes of the mouse mucosa. **A** The UMAP density plot of the condition-specific invariant embedding by scDisco based on interferon alpha stimulation (interferon alpha) and no stimulation (none) as the two distinct conditions. **B** The heatmap of the average expression matrix of condition-specific genes between interferon alpha and none conditions, normalized to a range of 0–1, reveals the relative expression levels. Deeper shades of blue indicate higher average gene expression values

To verify the capability of scDisco in effectively disentangling condition-specific factors from shared biological effects, we conducted experimental validations on all six real datasets: human lung, mouse mucosa, human ductal, human pancreas2, human pancreas, and human epithelium. The UMAP plots depicting the condition-specific invariant representations acquired through encoding [Figs. 6A, 7A, S13, S14, S15 and S16 in the Supplementary Figures of Additional file 1] reveal the discriminative prowess of scDisco in effectively discerning various condition categories. It is worth noting that the darker colors observed in the plots correspond to higher Gaussian kernel density estimates of the computed embeddings, indicating a denser concentration of data points in those corresponding regions.

By applying scDisco to the human lung dataset, we successfully identified condition-specific genes. Figure 6B shows that among the top 15 genes associated with the 62-year condition, 10 genes, such as MIR4435-2HG and NFATC3, exhibit abundant expression in this condition, with a significant number showing high expression levels. Previous research has demonstrated the involvement of MIR4435-2HG in promoting tumor cell proliferation, invasion, and apoptosis resistance, linking it to various types of cancer [42]. Furthermore, studies indicate that the risk of lung cancer increases in COPD patients compared to non-COPD individuals, and the risk further rises with the progression of COPD [43]. Increased expression of NFATC3 [44] in lung tissue of COPD patients promotes the production of inflammatory cytokines, aggravating inflammatory responses. The expression level of NFATC3 positively correlates with the severity of COPD. NFATC3 serves as a key transcription factor connecting chronic inflammation and tissue damage induced by tobacco smoke breathing in. It is noteworthy that the suppression of NFATC3 activity holds promise for improving the inflammatory response triggered by tobacco smoke exposure.

Given that the 62-year-old donor was diagnosed with chronic obstructive pulmonary disease (COPD), it is reasonable to consider the specific genes associated with the 62-year condition play a crucial role in the pathogenesis of this disease. To visually demonstrate this, we generated a differential expression matrix plot (Fig. 6C) encompassing all 45 genes linked to the three condition categories. Both the 58-year and 69-year donors were disease-free, and thus, the differential genes compared to the 62-year diseased donor are visually distinguished with darker colors of yellow in the lower-right corner and the middle region. These two condition categories (58-year and 69-year) share condition-associated genes with the 69-year category.

Furthermore, we employed the mouse mucosa dataset, with the stimulation of interferon alpha (IFN-$\alpha$) serving as the condition, to identify genes associated with stimulation. Figure 7B demonstrates that out of the 15 genes identified by scDisco, 11 genes, such as Spint1 and FGF9, exhibited elevated expression levels, indicating a robust association with IFN-$\alpha$. These findings suggest that these specific genes undergo alterations when mice are exposed to interferon alpha stimulation. Previous studies have proposed that IFN-$\alpha$ may induce the upregulation of Spint1 expression, thereby inhibiting the aging process in the skin [45]. Moreover, IFN-$\alpha$ has been shown to upregulate FGF9 mRNA expression in dendritic cells derived from mouse bone marrow, facilitating further understanding of IFN-$\alpha$’s role in immune regulation [46].

Subsequently, we validated scDisco using the human ductal dataset, comprising two groups of donors: individuals with primary pancreatic ductal adenocarcinoma (PDAC) tumors (T) and healthy individuals (N). Notably, Additional file 1: Fig. S13B showcases that scDisco successfully identified 12 out of the 15 genes (PKHD1L1, XIRP1, CTSV, MAGEA6, PIP, GFI1, CDH19, MEOX1, CNR2, CA12, GNAT3, KLK8, ITGB6) as exhibiting significantly high expression levels in association with primary PDAC tumors, indicating their significant correlation with the disease. Previous studies have shown that CTSV is primarily expressed in the stromal tissue surrounding PDAC tumor cells, and its elevated expression is associated with poor prognosis in PDAC patients, making it a potential biomarker and treatment target for prognostic assessment [47]. Increased expression of MAGEA6 suppresses autophagy in PDAC cells, promoting disease progression and establishing it as a potential therapeutic target [48]. Furthermore, GFI1 is linked to drug resistance in PDAC, as high Gfi-1 expression can enhance tolerance to chemotherapy drugs in PDAC treatment, making it a potential target for overcoming drug resistance [49].

In addition, we further validated the effectiveness of scDisco on three real datasets: human pancreas2, human pancreas, and human epithelium. For the human pancreas2 dataset, as illustrated in Additional file 1: Fig. S14B, scDisco successfully identified 9 out of the 15 genes that exhibited significantly elevated expression levels in connection with type II diabetes mellitus, underscoring their strong correlation with the disease. Similarly, in the human pancreas dataset, as shown in Additional file 1: Fig. S15B, scDisco also identified 9 out of the 15 genes displaying significantly high expression levels associated with type II diabetes mellitus. For the human epithelium dataset, as depicted in Additional file 1: Fig. S16B, scDisco effectively identified 10 out of the 15 genes displaying significantly elevated expression levels associated with childhood onset asthma, highlighting their substantial association with the disease. These satisfactory results were consistently observed across all six real datasets.

### Computational time and memory usage

We conducted comprehensive experiments to compare the performance of scDisco with nine other methods in terms of time consumption and memory utilization, measuring the time and memory usage for a single, non-sampled experiment on each dataset for every method. The evaluations were carried out on six real datasets: human pancreas2, human lung, human pancreas, mouse mucosa, human epithelium, and human ductal. These datasets range in size from 2125 to 57,530 cells.

**Fig. 8:**
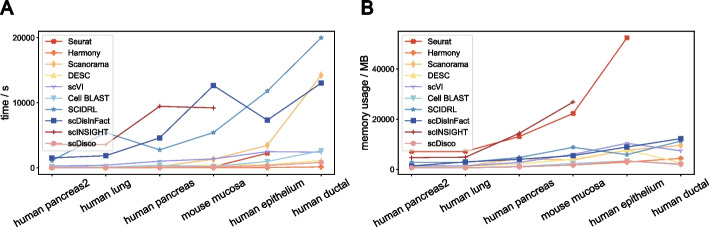
The time consumption and memory utilization across six real datasets. **A** The line chart of the time consumption of ten methods on the six real datasets. **B** The line chart of the memory utilization of ten methods on the same datasets. Each line represents a method, with the x-axis representing the datasets arranged in ascending order of cell numbers

Figure 8A reveals that scDisco exhibits a distinct advantage in terms of time efficiency. As the dataset size increases, scDisco demonstrates near-linear time consumption growth with a relatively gentle rate, highlighting its scalability. Notably, methods that process the human pancreas2 dataset with a larger sample size require more time. However, due to excessive memory usage and prolonged processing time, scINSIGHT faced limitations in handling the human epithelium and human ductal datasets, preventing their inclusion in the presented chart. Regarding memory utilization (Fig. 8B), scDisco demonstrates a linear increase along with the dataset size for all datasets except human ductal, where Cell BLAST exhibits even lower memory consumption. Regrettably, due to Seurat’s substantial memory usage on the human ductal dataset, it could not be included in the analysis.

## Discussion

In this article, we propose the scDisco, an innovative method designed for batch effect removal in scRNA-seq data. We benchmark it against nine other batch effect removal methods on both simulated and real datasets. By decoupling the preprocessed scRNA-seq expression matrix annotated with batch labels, we obtain shared biological representations and condition-specific biological representations. This enables us to retain condition specificity while removing batch effects from the biological representations. Additionally, by applying gradient backpropagation to condition-specific representations, scDisco facilitated the identification of genes associated with specific conditions, thereby providing valuable insights into disease biology. For instance, we have observed a compelling positive correlation between NFATC3 and the severity of COPD. We extensively validated the batch effect removal capability of scDisco on a simulated dataset and six real datasets. Furthermore, we evaluated scDisco’s performance in uncovering condition-specific genes across six real datasets. The experimental results demonstrated that scDisco exhibits robustness compared to existing methods, as it not only removes batch effects but also retains batch-specific cell types to a certain extent. In terms of computational performance, both time consumption and resource utilization of scDisco exhibit a slow linear increase as the number of cells increases, thus providing advantages across the six real datasets.

In contrast to conventional autoencoder networks employed in existing frameworks for obtaining low-dimensional representations, scDisco leverages the power of a variational autoencoder (VAE) network to achieve superior cell embedding in low-dimensional space. Moreover, scDisco effectively guides batch effect removal by incorporating batch information during the initial input stage, leading to more stable network training compared to directly decoupling batch noise. Considering the non-orthogonal relationship between condition biological effects and cellular biological variations, scDisco employs a shared encoder in the initial phase and condition-specific DSBN layers later on to better eliminate shared information between conditions, ultimately facilitating the extraction of condition biological effects across specific condition domains. Additionally, scDisco preprocessing picks out highly variable genes, aiding the model in capturing richer information for condition-specific data. Proper parameter selection during the neural network training process, within a certain range, enhances scDisco’s performance. While these steps assist in integrating scRNA-seq data, theoretically, perfect removal of batch effects cannot be guaranteed. Aiming to further eliminate batch effects, scDisco can be improved tailored to practical applications. For example, utilizing maximum mean discrepancy (MMD) loss can match the distributions between two batch datasets that share many common cell types [50]. For datasets with minimal shared cell types, employing discriminative loss can prevent variables after integration from distinguishing their respective batches, which should be careful to avoid mixing batch-specific cell types with other cells.

At the same time, it is important to note that even though scDisco successfully identifies genes associated with specific conditions, the set of condition-related genes it discovers may not encompass all relevant genes. Indeed, condition effects have significant correlation with cellular biology effects and do not exhibit a completely orthogonal relationship, making it extremely challenging to fully decouple condition effects unrelated to biology. Handling multiple confounding factors also introduces this challenge. The inter-correlation among these factors implies that disentangling these effects while maintaining condition-specific biological effects is a formidable task. However, by building upon the existing scDisco model, we can expand it to handle multiple different conditions. By establishing hierarchical relationships in the condition encoders for various factors, the model automatically identifies the construction of encoders based on the actual input factors. Subsequently, we can introduce MMD loss to align the distributions of the same conditions and create a distinct separation between the distributions of different conditions. Future research will endeavor to explore methods that more comprehensively disentangle condition-specific effects from biological effects.

While scDisco is developed specifically for batch effect removal in scRNA-seq data, the overall VAE framework, decoupling principles, and condition-specific DSBN layers can be extended to modeling other types of data that can be collected using existing technologies, such as scATAC-seq and spatial transcriptomics [51]. Simultaneously, by introducing a time-dependent factor to scDisco and incorporating time-dependent embeddings to capture temporal progression, the model can effectively handle time-dependent data, providing promising directions for future applications. Leveraging the scalability of scDisco, it is also possible to perform condition-specific gene selection through the integration of multiple omics data. This extension allows us to disentangle more accurate condition-specific information using scDisco and gain a deeper understanding of the regulatory mechanisms underlying gene expression or protein synthesis.

## Conclusions

In conclusion, this manuscript introduces scDisco, a novel method for batch effect removal. It utilizes a variational autoencoder (VAE) network to enhance low-dimensional cell embeddings, guiding batch effect removal by incorporating batch information at the initial input stage. Initially, we employ a shared encoder, which helps to retain batch-specific cell types to a certain extent while removing batch effects. By decoupling and employing condition-specific DSBNs, scDisco derives shared biological representations and more specific condition representations, enabling the identification of condition-specific genes that provide valuable insights into disease biology. Additionally, scDisco features reduced network complexity, which aids both in time consumption and resource utilization, increasing in a slow linear fashion as the number of cells increases.

### Supplementary Information

**Additional file 1** is a supplementary pdf file that contains method details, evaluation metrics and results along with relevant figures and tables omitted from the manuscript.

## Data Availability

The code is freely accessible at the website [52]: https://github.com/Victory-LRJ/scDisco. The real datasets supporting the conclusions [53] of this article are available in https://drive.google.com/drive/folders/1OCN6UmUsM98CpsecpbmQZsXmS0HKcB4k?usp=drive_link.

## Abbreviations

ARI: Adjusted rand index
ASW: Average silhouette width
BASW: Batch-wise average silhouette width
BN: Batch normalization
CASW: Cell-wise average silhouette width
DSBNs: Domain-specific batch normalization layers
ELBO: Evidence lower bound
GAN: Generative adversarial network
ID: Identification
KL: Kullback-leibler
MMD: Maximum mean discrepancy
MNN: Mutual nearest neighbors
NMF: Non-negative matrix factorization
NMI: Normalized mutual information
PDAC: Primary pancreatic ductal adenocarcinoma
ReLU: Rectified linear unit
scATAC-seq: Single-cell assay for transposase-accessible chromatin using sequencing
scRNA-seq: Single cell RNA sequencing
UMAP: Uniform manifold approximation and projection
VAE: Variational autoencoders
