# Supplementary material for: Integration of scRNA-seq data by disentangled representation learning with condition domain adaptation
Source: BMC Bioinformatics. 2024 Mar 16;25:116. doi: 10.1186/s12859-024-05706-9 (PMC10944609; doi:10.1186/s12859-024-05706-9)
Supplement: Supplementary file 1 — Additional file 1 is a supplementary pdf file that contains method details, evaluation metrics and results along with relevant figures and tables omitted from the manuscript. [file 12859_2024_5706_MOESM1_ESM.pdf]

Additional file 1 for "scDisco: Integration of scRNA-seq Data by Disentangled Representation Learning with Condition Domain Adaptation"

Renjing Liu[1†], Kun Qian[1†], Xinwei He[1], Hongwei Li[1*]

[1]School of Mathematics and Physics, China University of Geosciences (Wuhan), Wuhan, 430074, China.

*Corresponding author(s). E-mail(s): hwli@cug.edu.cn;
Contributing authors: liurenjing@cug.edu.cn; kun_qian@foxmail.com; hxinwei98@gmail.com;
†These authors contributed equally to this work.

1 Supplementary Tables

Table S1 The dimension setting of common-bio encoder

No. of cells	Dims
$n \leq 5000$	20
$5000 < n \leq 20000$	22
$20000 < n \leq 30000$	28
$n \geq 30000$	30

Table S2 ARI values given different values of λ and μ on the simulated data. The parameter λ controls the strength of the KL divergence, which measures the similarity between the learned distribution in the latent space and the assumed Gaussian distribution. We set $\lambda < 1$ to focus the model more on improving the quality of the latent space representation rather than forcing the model to match a specific prior distribution and generate samples that satisfy certain prior assumptions. The parameter μ in the overall loss function controls the relative weight between the variational autoencoder loss. Since condition-specific biological effects in actual biology are smaller than the effects shared across cells, we evaluated the impact of μ values in the range of $\mu < 1$. To achieve optimal results for these two parameters λ and μ, we performed an extensive search within the respective ranges. We evaluated λ values of 0.1, 0.01, 0.001, and 0.0001, and μ values of 0.1, 0.01, 0.001, and 0.0001, while keeping other parameters constant and conducted experiments on simulated data and six real datasets.

μ \ λ	0.1	0.01	0.001 (default)	0.0001
0.1	0.12	0.73	0.82	0.92
0.01	0.16	0.77	0.99	0.98
0.001	0.18	0.73	**1.00**	0.99
0.0001	0.10	0.78	0.92	0.87

Table S3 ARI values given different values of λ and μ on the Human pancreas2 dataset.

μ \ λ	0.1	0.01	0.001 (default)	0.0001
0.1	0.24	0.28	0.89	0.88
0.01	0.16	0.30	0.87	0.87
0.001	0.24	0.29	**0.93**	0.91
0.0001	0.17	0.30	0.89	0.88

Table S4 ARI values given different values of λ and μ on the Human lung dataset.

μ \ λ	0.1	0.01	0.001 (default)	0.0001
0.1	0.02	0.05	0.48	**0.66**
0.01	0.01	0.25	**0.66**	0.56
0.001	0.01	0.22	0.65	0.63
0.0001	0.01	0.23	0.64	0.60

Table S5 ARI values given different values of λ and μ on the Human pancreas dataset.

λ / μ	0.1	0.01	0.001 (default)	0.0001
0.1	0.02	0.33	0.69	0.89
0.01	0.08	0.30	0.89	**0.91**
0.001	0.09	0.30	0.90	0.90
0.0001	0.08	0.30	**0.91**	**0.91**

Table S6 ARI values given different values of λ and μ on the Mouse mucosa dataset.

λ / μ	0.1	0.01	0.001 (default)	0.0001
0.1	0.02	0.55	0.72	0.43
0.01	0.04	0.37	0.63	0.64
0.001	0.03	0.35	**0.73**	0.68
0.0001	0.04	0.40	0.68	0.64

Table S7 ARI values given different values of λ and μ on the Human epithelium dataset.

λ / μ	0.1	0.01	0.001 (default)	0.0001
0.1	0.09	0.13	0.04	0.48
0.01	0.09	0.49	0.18	0.38
0.001	0.08	0.47	**0.56**	0.52
0.0001	0.09	0.51	0.52	0.47

Table S8 ARI values given different values of λ and μ on the Human ductal dataset.

λ / μ	0.1	0.01	0.001 (default)	0.0001
0.1	0.30	0.25	0.34	0.76
0.01	0.15	0.55	0.28	0.74
0.001	0.29	0.52	**0.86**	0.81
0.0001	0.14	0.54	0.80	0.74

Table S9 The average ARI values given different values of λ and μ on seven datasets.

μ \ λ	0.1	0.01	0.001 (default)	0.0001
0.1	0.12	0.33	0.57	0.72
0.01	0.10	0.43	0.64	0.73
0.001	0.13	0.41	**0.80**	0.78
0.0001	0.09	0.44	0.77	0.73

Table S10 Introduction of nine comparison methods. scVI is implemented using the exclusive virtual environment scvi-tools; Cell BLAST is implemented using the exclusive virtual environment cb; the source code for SCIDRL can be downloaded from its GitHub repository.

Methods	Version	Software	Download URLs	No. of citations
Seurat	4.3.0	R4.1.3	https://cran.r-project.org/web/packages/Seurat/index.html	7660
Harmony	0.0.9	Python-3.8.8	https://github.com/immunogenomics/harmony	2510
Scanorama	1.7.3	Python-3.8.8	https://github.com/brianhie/scanorama	489
DESC	2.1.1	Python-3.8.8	https://eleozzr.github.io/desc/	289
scVI	0.14.6	Python-3.9.16	https://github.com/scverse/scvi-tools	1055
Cell BLAST	0.5.0	Python-3.9.16	https://github.com/gao-lab/Cell_BLAST	73
SCIDRL	NA	Python-3.8.8	https://github.com/guott15/SCIDRL.git	1
scDisInFact	0.1.0	Python-3.8.8	https://github.com/ZhangLabGT/scDisInFact	0
scINSIGHT	0.1.4	R-4.1.3	https://cran.r-project.org/web/packages/scINSIGHT/index.html	8

Table S11 Pre-processing steps and parameter settings for the nine comparison methods.

Methods	Data pre-processing	Highly variable genes required	Parameters
Seurat	1. Normalization. Gene expression values for each cell were divided by the total number of transcripts and multiplied by 10000. These values were then natural-log transformed using log1p before further down-stream analyses.<br>2. Selection of highly variable genes.	2000 (default)	default
Harmony	1. Normalization. Use standard log counts-per-10000 transformation.<br>2. Perform PCA (default 20).	No	default
Scanorama	1. Filtering cells and genes. Genes that are not expressed in fewer than one cell and cells with less than 600 expressed genes are filtered out.<br>2. Normalization. Perform l_2-normalized the expression values for each cell for scale-invariant comparison.	No	default
DESC	1. Filtering cells and genes. Genes that are not expressed in fewer than 3 cells and cells with less than 200 expressed genes are filtered out.<br>2. Normalization. each cell by total counts of all genes, so that every cell has the same total count after normalization (10000 by default).<br>3. Logarithm transformation. transformed to a natural log scale.<br>4. Selection of highly variable genes.<br>5. Scaling. When batch information is provided, gene expression standardization is performed across cells in each batch separately.	1000 (default)	default
scVI	1. Filtering cells and genes. Genes that are not expressed in fewer than 3 cells and cells with less than 3 expressed genes are filtered out.<br>2. Normalization. The gene expression of each cell is normalized by its library size factor. The library size factor is defined as the total count in the cell divided by the median total count of all cells.<br>3. Logarithm transformation. The normalized data is log-transformed.<br>4. Selection of highly variable genes. Perform this gene selection using the Scanpy pipeline and obtain variable genes from each dataset and take their intersections.	2000 (default)	default
Cell BLAST	1. Selection of highly variable genes. Select most informative genes that cover most of the data variation on different "batches" of the data, and merge selected genes from individual "batches" via a voting strategy.<br>2. Performing data normalization or gene subsetting externally beforehand is not recommended.	By default, genes selected in 50% of the "batches" will be preserved, which is controlled by the parameter.	default
SCIDRL	1. Normalization. The input gene expression levels of each cell are first normalized to the same scale of 10000, which is followed by log transformation.<br>2. Selection of highly variable genes. Use the 'FindVariableFeatures' of Seurat toolkit with 'vst' for parameter 'method' to select the top 1000 highly variable genes (HVG) for each batch.	1000 (default)	default
scDisInFact	scDisInFact can be trained directly on the raw scRNA-seq dataset, no pre-processing step is required before running scDisInFact.	No	default
scINSIGHT	1. Normalization. Scale the counts such that each cell has a total of 10000 reads or UMIs and perform log-transformation on the scaled values.<br>2. Selection of highly variable genes. Identify 2000 highly variable genes.	2000 (default)	default

Table S12 The dispersion of the same cell type on simulated data of scDisco and nine comparison methods for six cell types. For cells $i \in c(i = 1, 2, , N)$ belonging to the same cell type c, assuming the cluster labels after integration are $c_j(j = 1, 2, , J)$. Then the cluster labels were sorted by cluster size in descending order, with the largest cluster labeled as c'_j. We calculated the proportion of cells in the largest cluster, denoted as $p_c = count(c_{j'})/N$, which ranges between [0, 1]. A higher score indicates that most cells of type c remain in the same cluster after integration, suggesting that they are still the same cell type. If the proportions in the largest cluster are generally low, it implies that cells of type c are distributed across multiple clusters, indicating dissimilarity after integration. When p_c is 1, it means that cells of type c_j are all assigned to the same cluster after integration, indicating no over-correction.

Methods	c1	c2	c3	c4	c5	c6
Seurat	1	1	1	0.52	0.91	0.55
Harmony	1	1	1	0.99	1	1
Scanorama	1	1	0.67	75	0.5	0.75
DESC	1	1	1	1	1	1
scVI	1	1	1	0.93	0.91	0.91
Cell BLAST	1	1	0.52	0.57	0.91	0.87
SCIDRL	0.33	0.33	0.33	0.26	0.25	0.25
scDisInFact	1	0.59	1	0.52	0.61	0.87
scINSIGHT	1	1	1	1	1	1
scDisco	1	1	1	0.99	1	1

Table S13 F1 scores for scDisco and nine comparison methods on all seven datasets. Bold indicates the top-ranked, and underline indicates the second-ranked. Nan represents missing value.

Methods	Simulation	Human pancreas2	Human lung	Human pancreas	Mouse mucosa	Human epithelium	Human ductal
Seurat	0.73	0.74	0.74	0.80	0.74	0.73	Nan
Harmony	0.83	0.78	0.76	0.79	0.75	0.71	**0.75**
Scanorama	0.66	0.52	0.56	0.57	0.64	**0.77**	0.62
DESC	**0.90**	0.80	0.76	0.75	0.61	0.67	0.64
scVI	0.72	0.71	0.70	0.73	0.72	0.69	0.70
Cell BLAST	0.73	0.80	0.75	0.78	0.75	0.70	0.71
SCIDRL	0.74	0.73	0.70	0.73	0.76	0.69	0.64
scDisInFact	0.62	0.56	0.57	0.58	0.75	0.64	0.58
scINSIGHT	0.84	**0.82**	0.76	**0.83**	0.72	Nan	Nan
scDisco	0.82	0.80	**0.79**	0.81	**0.78**	0.68	0.70

2 Supplementary Figures

2.1 Simulation Experiments

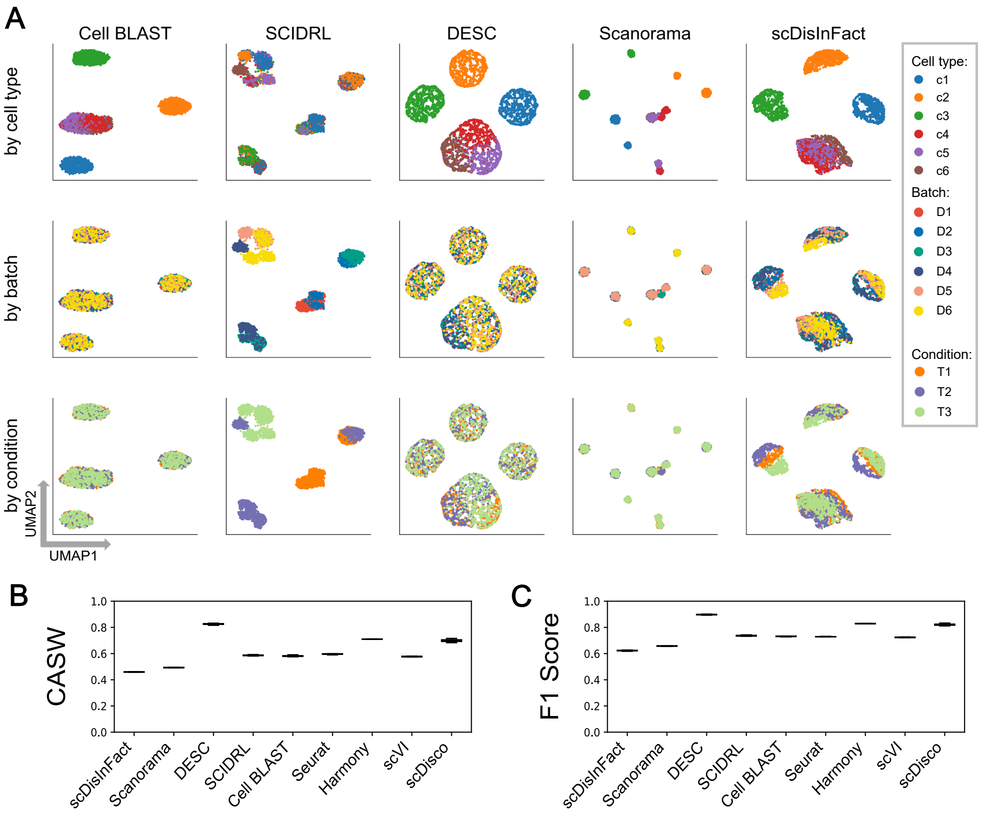

Fig. S1 The integrated comparation of the simulated data. **(A)** UMAP plots of the cell embeddings produced by Cell BLAST, SCIDRL, DESC, Scanorama and scDisInFact. Each point represents a cell, and each column represents a method, while each row corresponds to the UMAP plot with coloring based on true cell types, batch IDs, and condition IDs. **(B-C)** Boxplots of CASW and F1 Score values of each method by applying the nine integration methods to randomly selected subsamples of the complete simulated data.

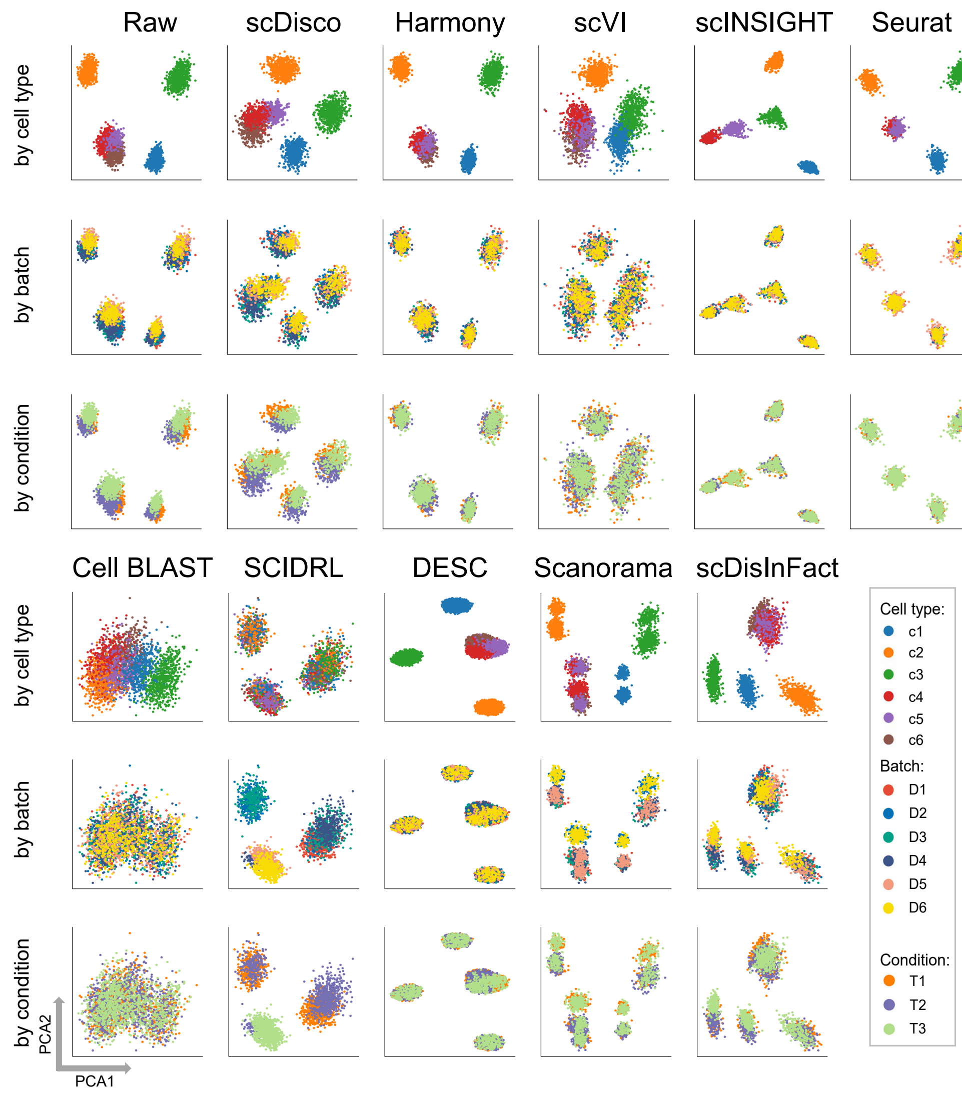

Fig. S2 The integrated comparation of the simulated data by PCA plots. The PCA plots of Raw and the cell embeddings produced by scDisco, Harmony, scVI, scINSIGHT, Seurat, Cell BLAST, SCIDRL, DESC, Scanorama, and scDisInFact. Each point represents a cell, and each column represents a method, while each row corresponds to the PCA plot with coloring based on true cell types, batch IDs, and condition IDs. The distances on the plot intuitively reflect the approximated distances between cells.

2.2 Identifies batch-specific cell types.

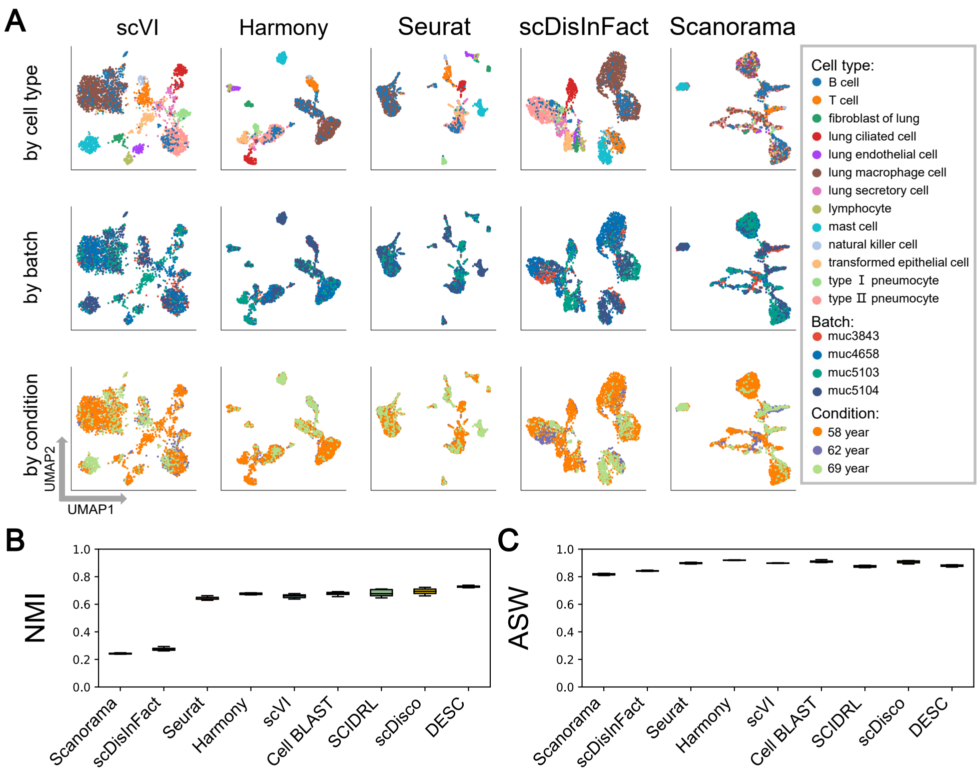

Fig. S3 The integrated comparation of the human lung dataset. **(A)** UMAP plots of the cell embeddings produced by scVI, Harmony, Seurat, scDisInFact, and Scanorama. Each point represents a cell, and each column represents a method, while each row corresponds to the UMAP plot with coloring based on true cell types, batch IDs, and condition IDs. **(B-C)** Boxplots of NMI and ASW values of each method by applying the nine integration methods to randomly selected subsamples of the complete human lung data.

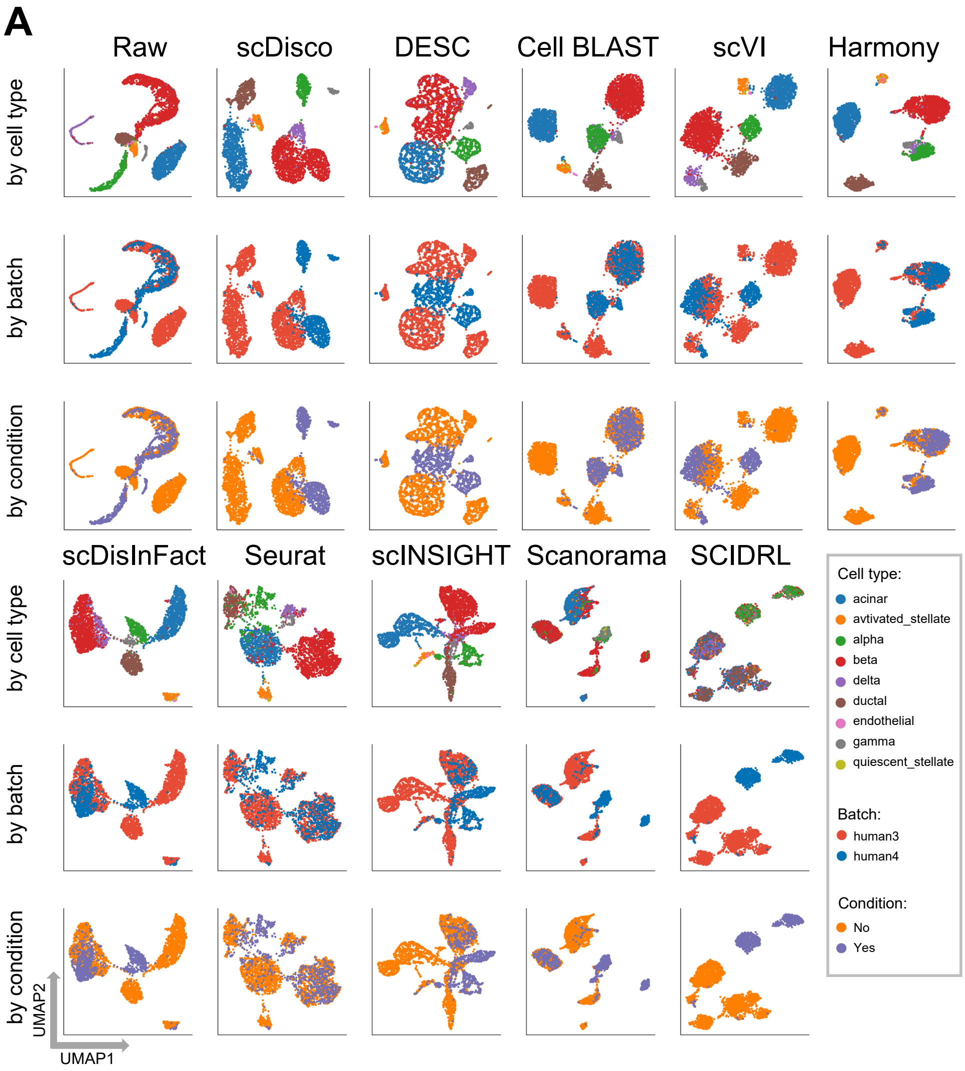
A
Raw
scDisco
DESC
Cell BLAST
scVI
Harmony
by cell type
by batch
by condition
scDisInFact
Seurat
scINSIGHT
Scanorama
SCIDRL
Cell type:
acinar
avtivated_stellate
alpha
beta
delta
ductal
endothelial
gamma
quiescent_stellate
Batch:
human3
human4
Condition:
No
Yes
UMAP2
UMAP1

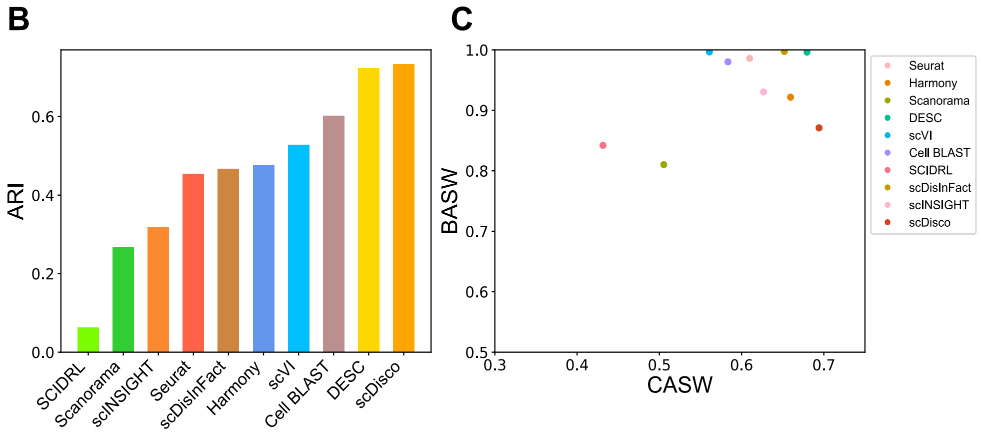

Fig. S4 The integrated comparation of the human pancreas-subset dataset. We utilized downsampling of the human pancreas to construct a specialized dataset with only one shared cell type. **(A)** The UMAP plots of Raw and the cell embeddings produced by scDisco, DESC, Cell BLAST, scVI, Harmony, scDisInFact, Seurat, scINSIGHT, Scanorama, and SCIDRL. Each point represents a cell, and each column represents a method, while each row corresponds to the UMAP plot with coloring based on true cell types, batch IDs, and condition IDs. **(B)** Bars of ARI values of each method by applying scDisco and nine comparison methods to the complete human pancreas-subset dataset. **(C)** CASW and BASW scores for scDisco and nine comparison methods on the human pancreas-subset dataset.

2.3 Human pancreas2

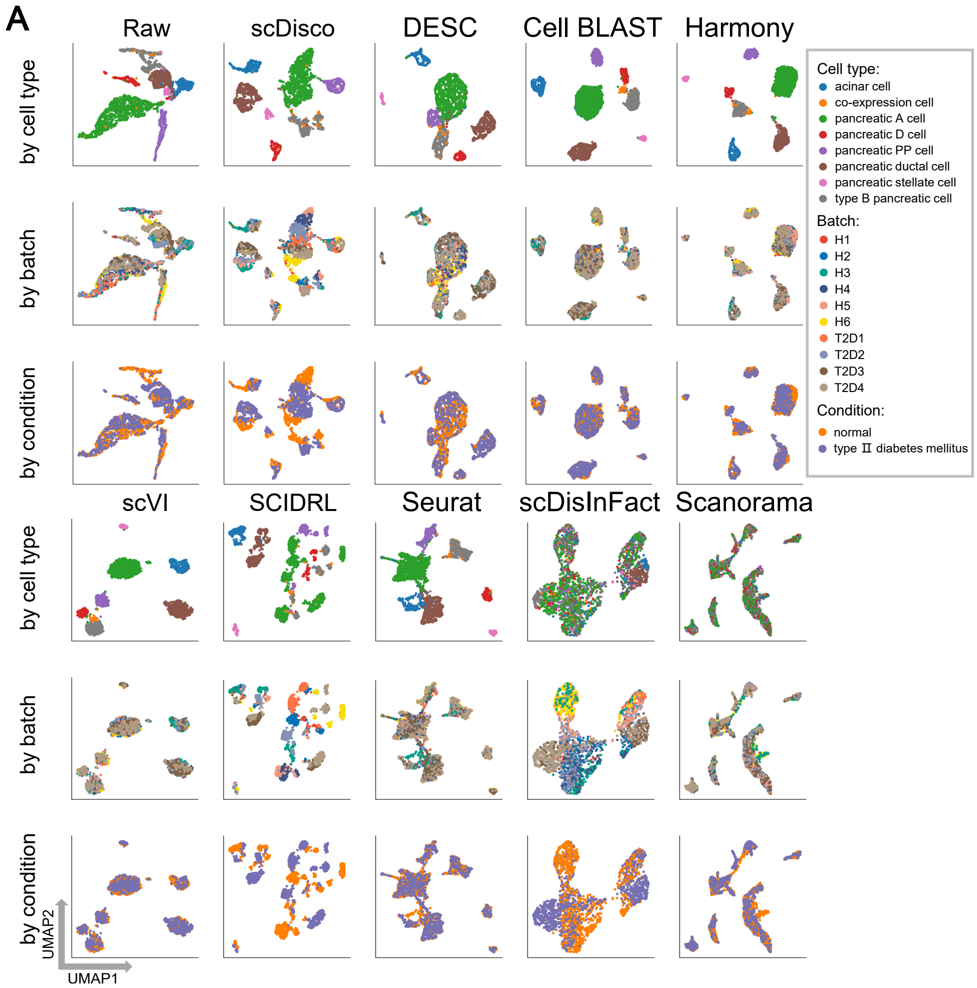

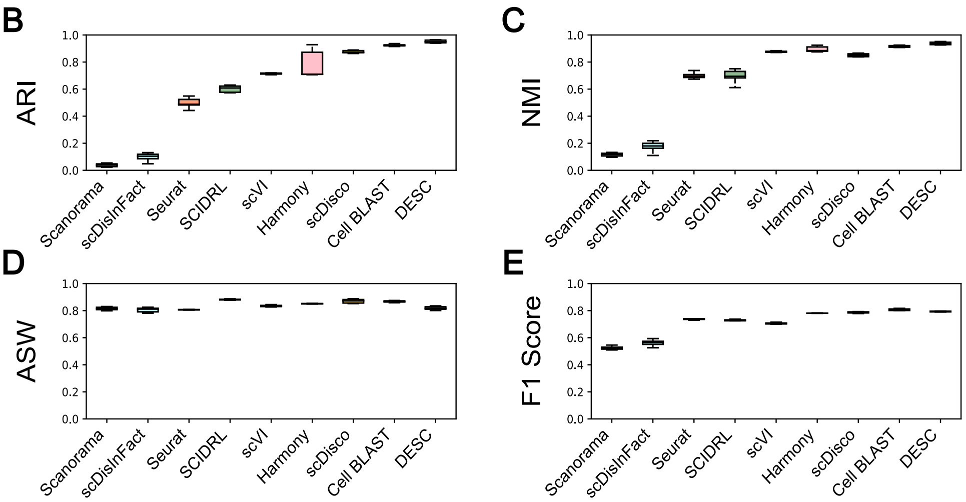

Fig. S5 The integrated comparation of the Human pancreas2 dataset. **(A)** UMAP plots of Raw and the cell embeddings produced by scDisco, DESC, Cell BLAST, Harmony, scVI, SCIDRL, Seurat, scDisInFact, and Scanorama. Each point represents a cell, and each column represents a method, while each row corresponds to the UMAP plot with coloring based on true cell types, batch IDs, and condition IDs. **(B-E)** Boxplots of ARI, NMI, ASW and F1 Score values of each method by applying the nine integration methods to randomly selected subsamples of the complete human pancreas data.

2.4 Human pancreas

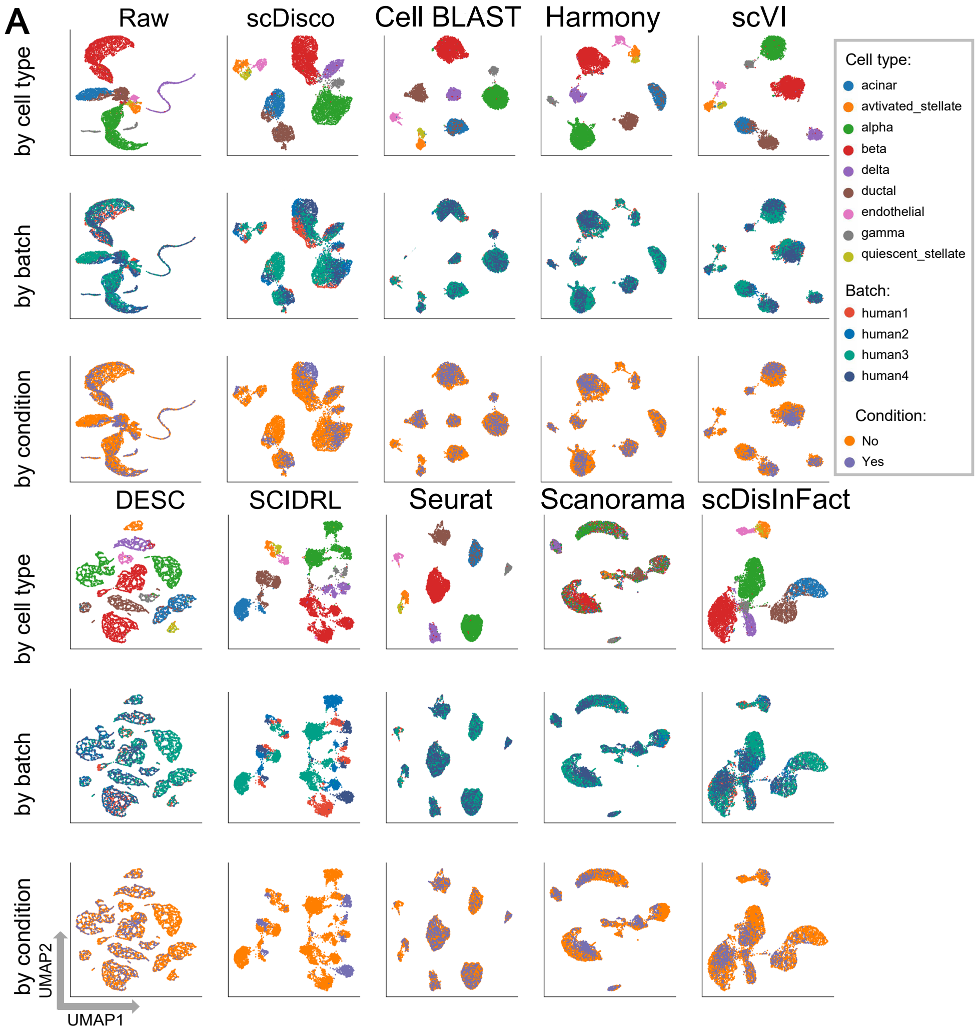

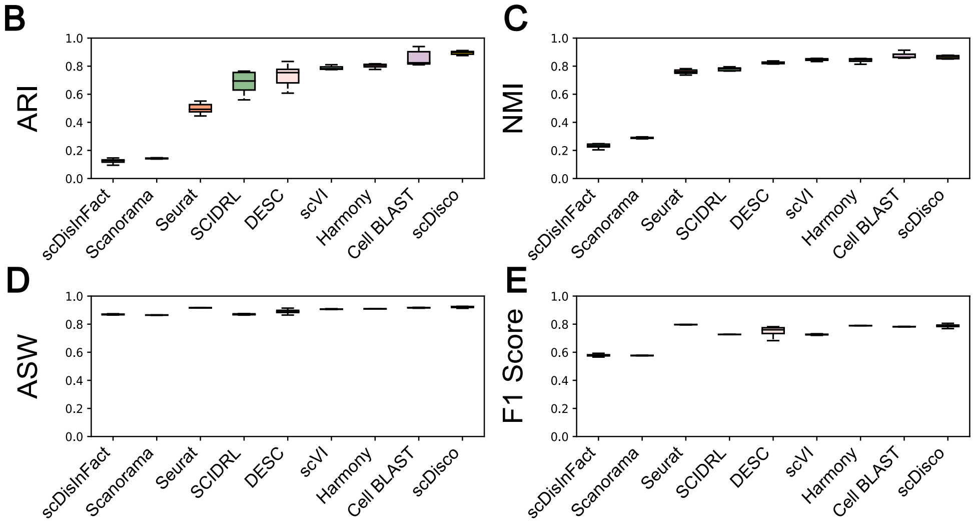

Fig. S6 The integrated comparation of the human pancreas dataset. **(A)** UMAP plots of Raw and the cell embeddings produced by scDisco, Cell BLAST, Harmony, scVI, DESC, SCIDRL, Seurat, Scanorama, and scDisInFact. Each point represents a cell, and each column represents a method, while each row corresponds to the UMAP plot with coloring based on true cell types, batch IDs, and condition IDs. **(B-E)** Boxplots of ARI, NMI, ASW and F1 Score values of each method by applying the nine integration methods to randomly selected subsamples of the complete human pancreas data.

2.5 Mouse mucosa

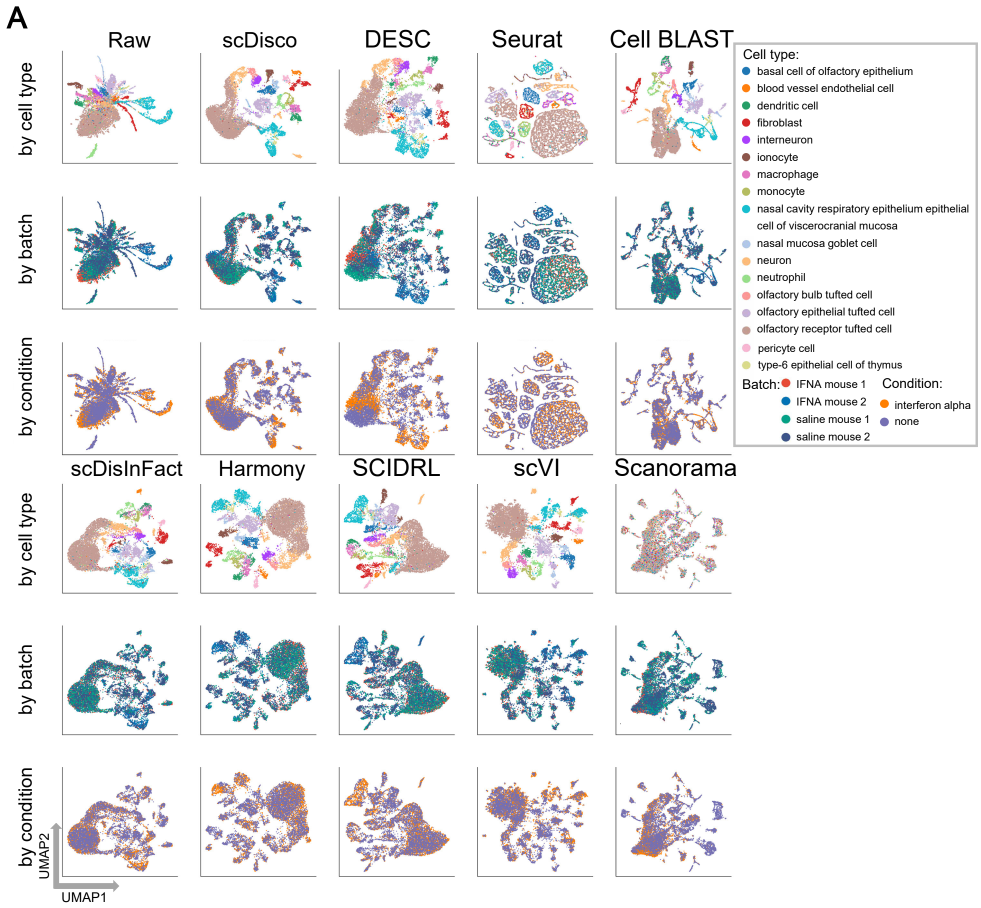

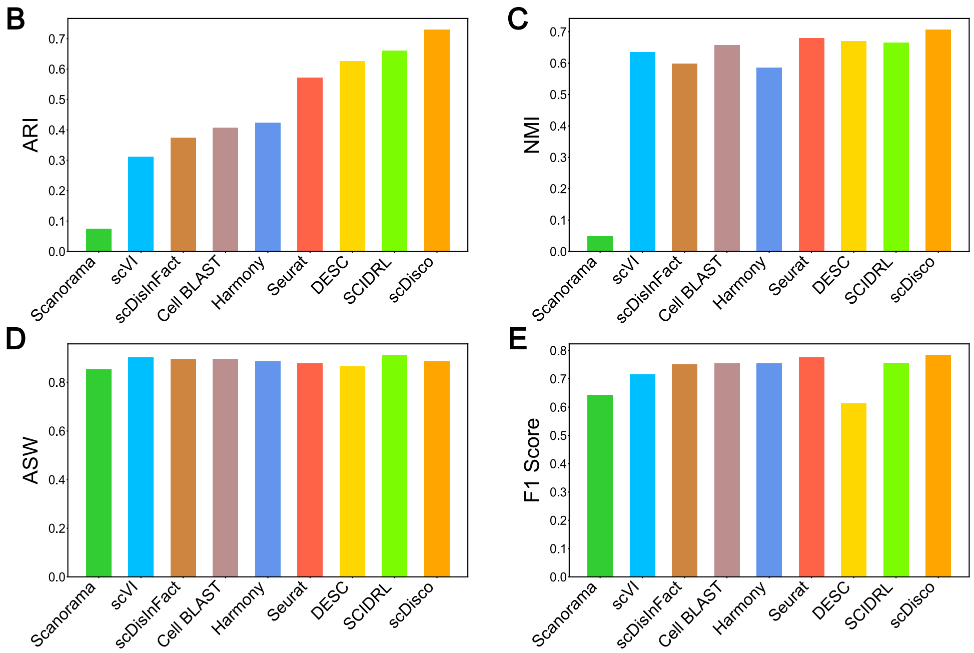

Fig. S7 The integrated comparation of the mouse mucosa dataset. **(A)** UMAP plots of Raw and the cell embeddings produced by scDisco, DESC, Seurat, Cell BLAST, scDisInFact, Harmony, SCIDRL, scVI and Scanorama. Each point represents a cell, and each column represents a method, while each row corresponds to the UMAP plot with coloring based on true cell types, batch IDs, and condition IDs. **(B-E)** Bars of ARI, NMI, ASW and F1 Score values of the eight integration methods of the complete mouse mucosa data.

2.6 Human epithelium

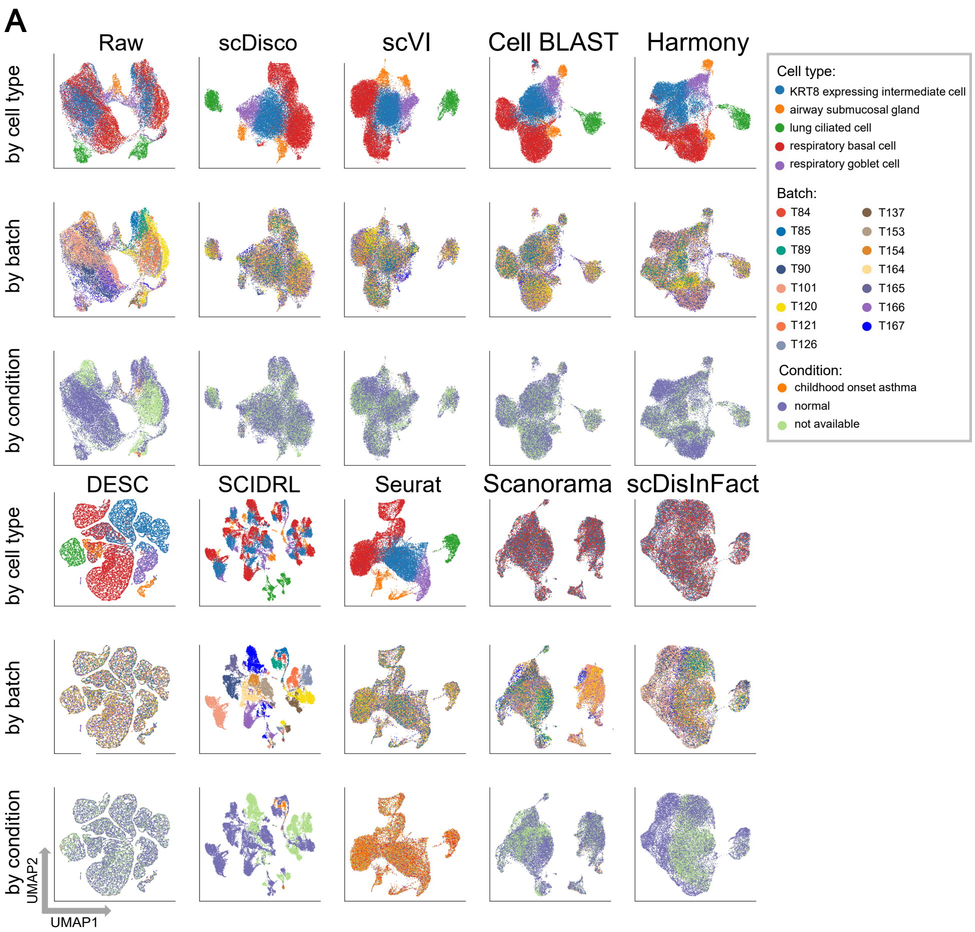

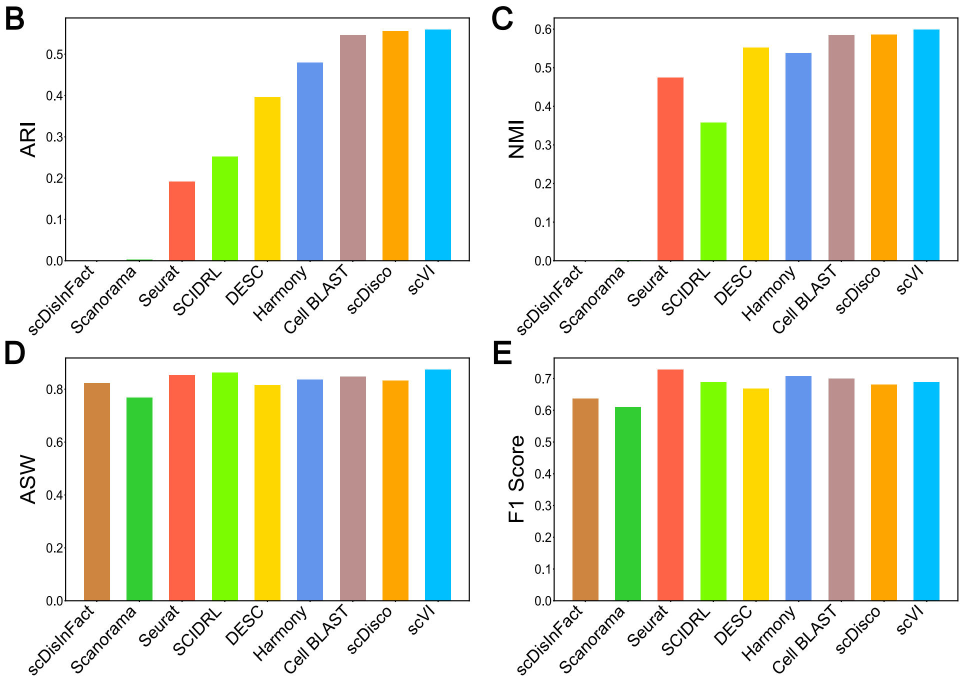

Fig. S8 The integrated comparation of the human epithelium dataset. **(A)** UMAP plots of Raw and the cell embeddings produced by scDisco, scVI, Cell BLAST, Harmony, DESC, SCIDRL, Seurat, Scanorama and scDisInFact. Each point represents a cell, and each column represents a method, while each row corresponds to the UMAP plot with coloring based on true cell types, batch IDs, and condition IDs. **(B-E)** Bars of ARI, NMI, ASW and F1 Score values of the eight integration methods of the complete human epithelium data.

2.7 Comparing scDisco with scINSIGHT

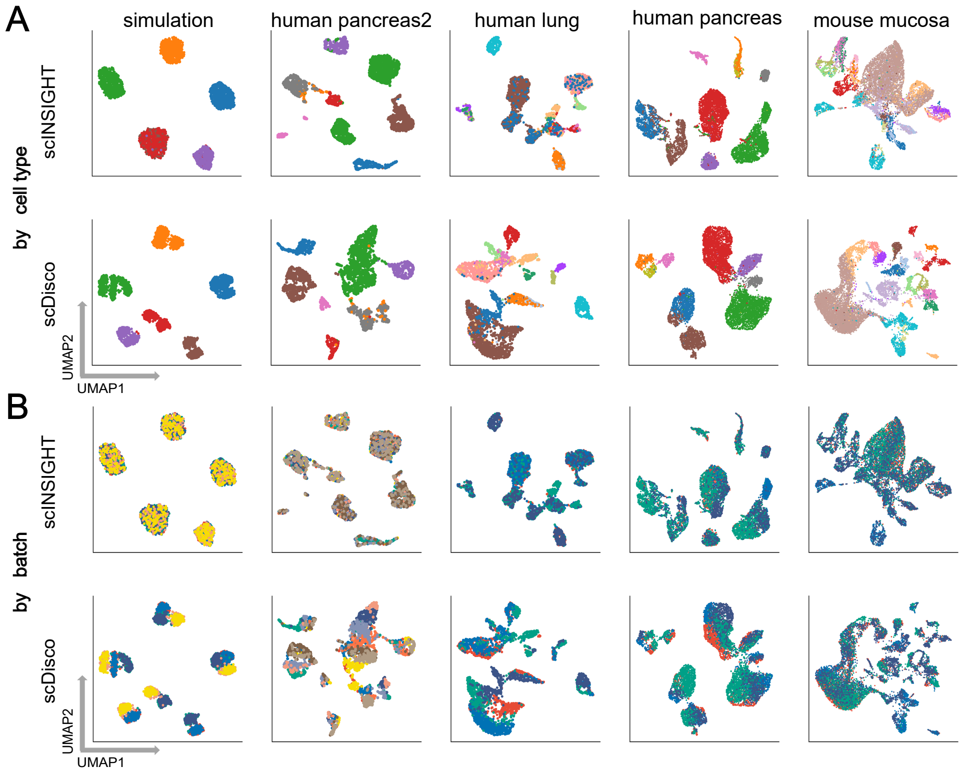

Fig. S9 The integrated comparation of the simulated data and four real datasets by scINSIGHT and scDisco. **(A)** UMAP plots of the cell embeddings produced by scINSIGHT and scDisco. Each point represents a cell, and each column represents a dataset, while each row corresponds to the UMAP plot with coloring based on true cell types. **(B)** UMAP plots same as shown in **(A)** but colored by batch IDs.

2.8 Human ductal

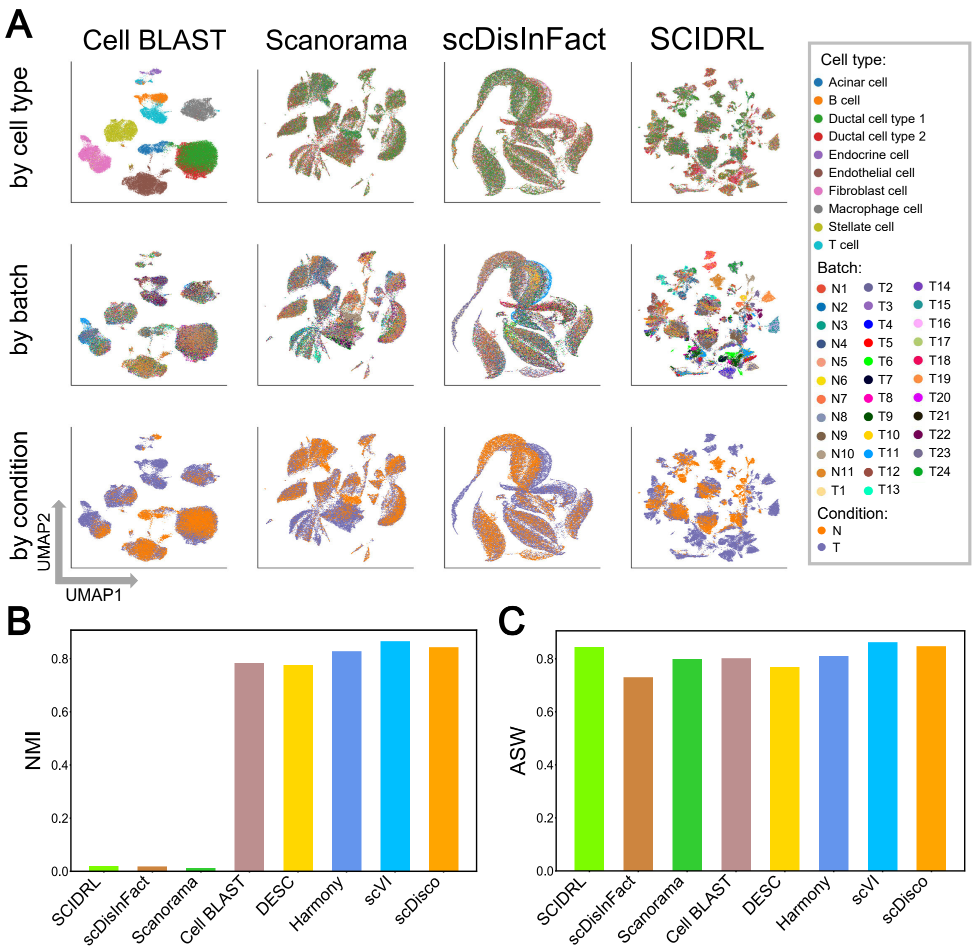

Fig. S10 The integrated comparation of the human ductal dataset. **(A)** UMAP plots of Raw and the cell embeddings produced by Cell BLAST, Scanorama, scDisInFact and SCIDRL. Each point represents a cell, and each column represents a method, while each row corresponds to the UMAP plot with coloring based on true cell types, batch IDs, and condition IDs. **(B-C)** Bars of NMI and ASW values of the eight integration methods of the complete human ductal data.

2.9 Evaluation of the batch effect removal on all datasets

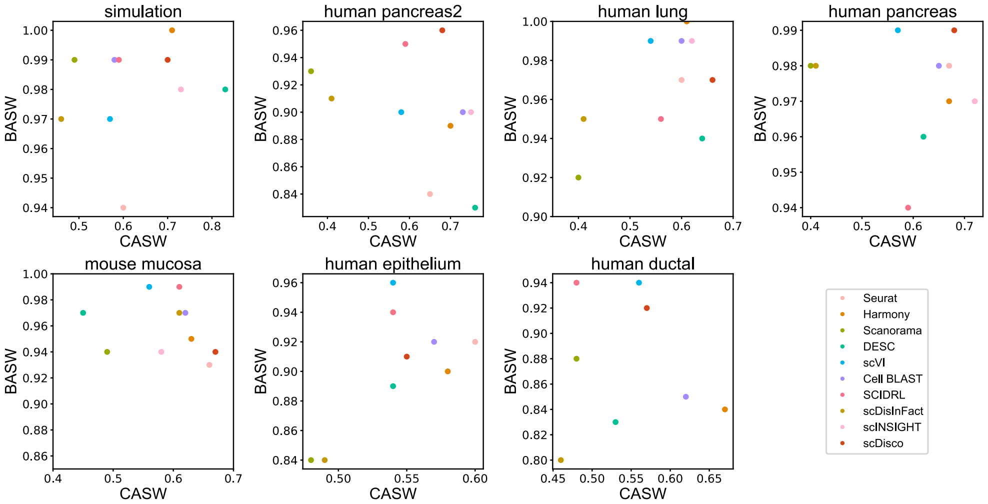

Fig. S11 CASW and BASW scores for scDisco and nine comparison methods on all seven datasets. Given that higher values for CASW and BASW indicate better performance, a model's overall effectiveness is better if it's located in the upper-right corner of the graph.

2.10 Sensitivity analysis

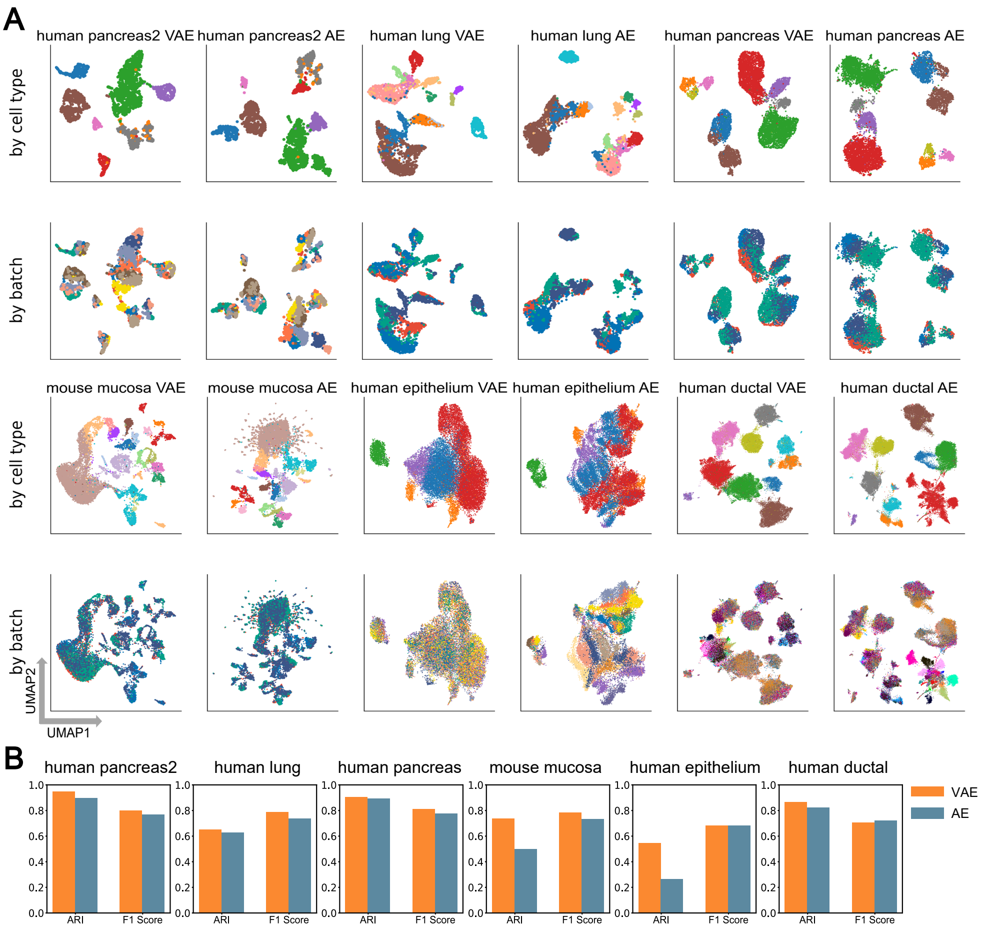

Fig. S12 Comparative analysis of scDisco's frameworks: VAE and AE, utilizing six real datasets. **(A)** UMAP plots of the cell embeddings produced by scDisco-VAE and scDisco-AE for six real datasets. Color-coded depiction according to cell type and batch ID. **(B-C)** Bars of ARI and F1 Score values of the scDisco-VAE and the scDisco-AE frameworks for six real datasets of the complete data. Subplots presented in the order of human pancreas2, human lung, human pancreas, mouse mucosa, human epithelium, and human ductal datasets.

2.11 Condition-specific genes

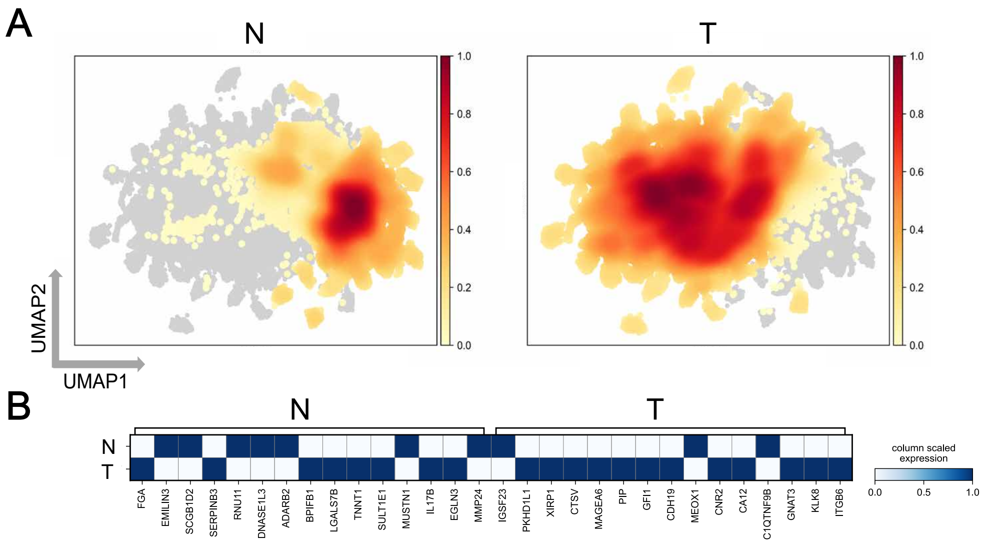

Fig. S13 Condition-specific genes of the human ductal. **(A)** The UMAP density plot of the condition-specific invariant embedding by scDisco based on primary PDAC tumor patients (T) and healthy donors (N) as the two distinct conditions. **(B)** The heatmap of the average expression matrix of condition-specific genes between tumor and normal conditions, normalized to a range of 0-1, reveals the relative expression levels. Deeper shades of blue indicate higher average gene expression values. To convert human Ensemble IDs to Gene symbols, we utilized version 4.2.2 of the 'org.Hs.eg.db' package[1] implemented in R 3.15.0. To convert mouse Ensemble IDs to Gene symbols, we utilized version 4.2.2 of the 'org.Mm.eg.db' package[2] implemented in R 3.15.0.

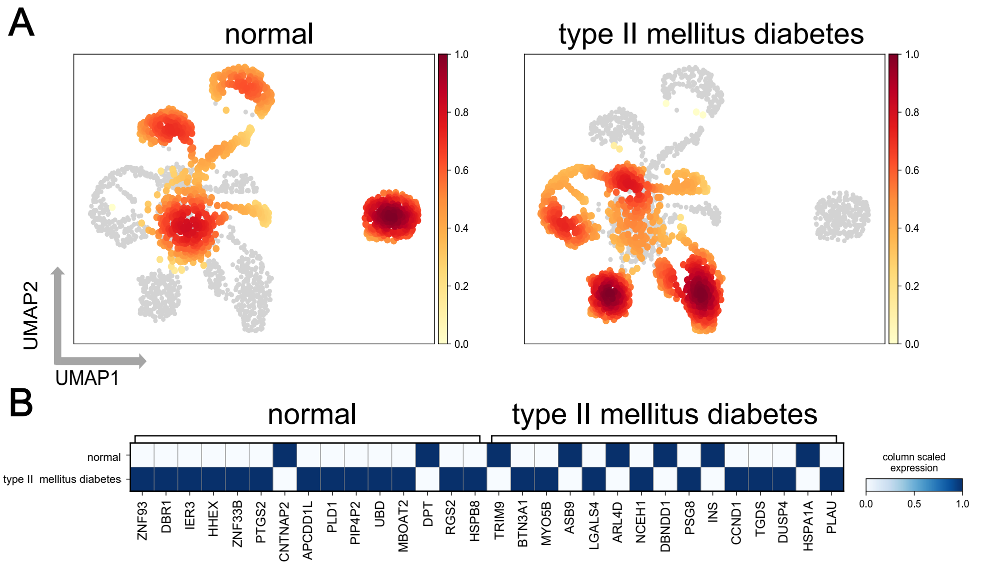

Fig. S14 Condition-specific genes of the human pancreas2. **(A)** The UMAP density plot of the condition-specific invariant embedding by scDisco based on normal donors (normal) and type II diabetes mellitus patients (type II diabetes mellitus) as the two distinct conditions. **(B)** The heatmap of the average expression matrix of condition-specific genes between normal and type II diabetes mellitus conditions, normalized to a range of 0-1, reveals the relative expression levels. Deeper shades of blue indicate higher average gene expression values.

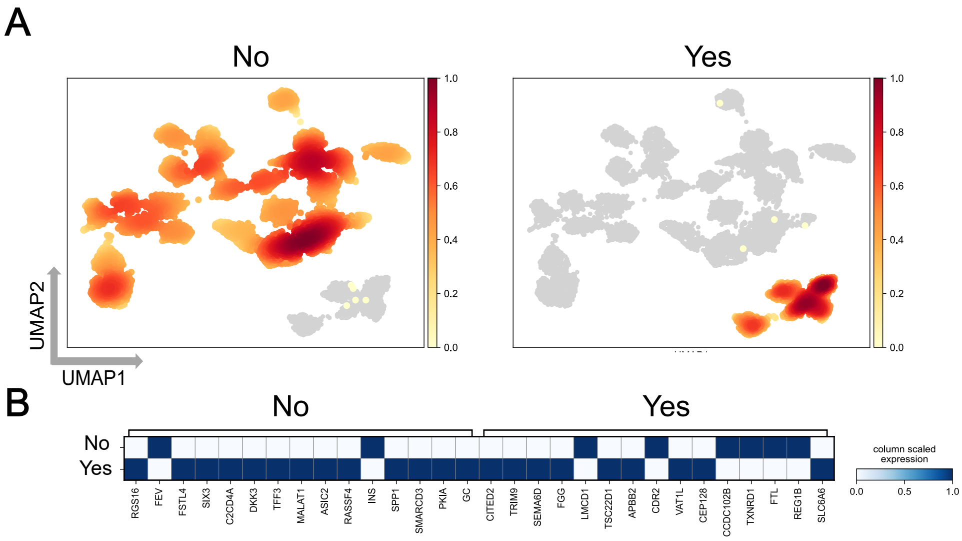

Fig. S15 Condition-specific genes of the human pancreas. **(A)** The UMAP density plot of the condition-specific invariant embedding by scDisco based on normal donors (N) and type II diabetes mellitus patients (Yes) as the two distinct conditions. **(B)** The heatmap of the average expression matrix of condition-specific genes between No and Yes conditions, normalized to a range of 0-1, reveals the relative expression levels. Deeper shades of blue indicate higher average gene expression values.

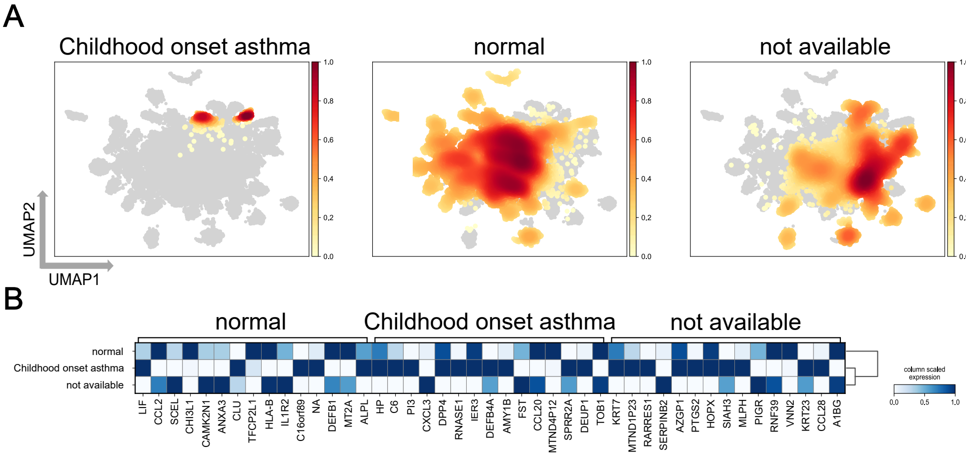

Fig. S16 Condition-specific genes of the human epithelium. **(A)** The UMAP density plot of the condition-specific invariant embedding by scDisco based on childhood onset asthma patients (childhood onset asthma), normal donors (normal) and donors without available disease information (not available) as the three distinct conditions. **(B)** The heatmap of the average expression matrix of condition-specific genes among childhood onset asthma, normal and not available conditions, normalized to a range of 0-1, reveals the relative expression levels. Deeper shades of blue indicate higher average gene expression values.

3 Supplementary Methods

3.1 Evaluation metrics

ARI. Adjusted Rand Index (ARI)[3] is a metric used to assess the overlap between two clusters, taking into account both correct clustering overlap and inconsistencies between the two clusters. It compares known cell type labels with the integrated clustering results, ranging from -1 to 1, where a value closer to 1 indicates better clustering performance.

NMI. Normalized Mutual Information (NMI)[4]measures the overlap between two clusters, focusing more on the global information interaction. It scales the average entropy of true cell types and integrated cluster labels. Comparing known cell type labels with the integrated clustering results, the NMI ranges from 0 to 1, where a value closer to 1 indicates better clustering performance.

ASW. Average Silhouette Width (ASW)[5] evaluates the batch correction effect by combining true cell type and batch labels. It assesses the relationship between the intra-cluster distance of a cell and the inter-cluster distance to its nearest cluster. ASW is obtained by averaging the silhouette widths of all cells in a set, ranging from -1 to 1.

Considering the absolute silhouette coefficient $s(i)$ on the batch label of each cell i, the formula for batch ASW is as follows:

$$ASW_{batchnorm} = |s(i)|. \tag{1}$$

This metric ranges from 0 to 1, where 0 represents good batch mixing, and values deviating from 0 indicate batch effects. Consequently, the batch ASW (BASW) can be calculated as follows:

$$BASW = 1 - ASW_{batchnorm}. \tag{2}$$

BASW ranges from 0 to 1, where 1 represents good batch mixing.

Taking into account the absolute silhouette coefficient $s(j)$ on the cell label of each cell j, the formula for normalized cell ASW(CASW) is as follows:

$$CASW = ASW_{celltypenorm} = \frac{s(j)+1}{2}. \tag{3}$$

CASW ranges from 0 to 1, where values closer to 1 indicate better batch mixing.

To ensure the stability of ASW scores, 90% of the original cell count is randomly sub-sampled, and this process is repeated 20 times to obtain 20 normalized cell type ASW scores and 20 normalized batch ASW scores. Next, for the combined evaluation of cell type purity and batch mixing, we compute the weighted average of batch and cell ASW scores:

$$ASW = 0.8 \cdot (1 - ASW_{batch\ norm}) + 0.2 \cdot ASW_{cell\ type\ norm}, \tag{4}$$

where ASW ranges from 0 to 1, with a higher ASW indicating better batch correction effectiveness.

To better assess the degree of batch mixing, the harmonic mean of batch and cell type ASW scores is calculated to obtain the F1 Score[6]:

$$F1\ Score = \frac{2(1 - ASW_{batch\ norm})ASW_{cell\ type\ norm}}{1 - ASW_{batch\ norm} + ASW_{cell\ type\ norm}}, \tag{5}$$

where a higher F1 Score (i.e., a higher batch ASW score and a higher cell type ASW score) indicates better batch correction effectiveness and a balance between batch effect removal and maintaining high cell purity.

In the section dedicated to cell clustering analysis, we utilize Z_bio for UMAP visualization and clustering metric calculations. Z_{bio} represents non-condition-specific

factors influencing shared biological effects among cells, capturing both cellular similarities and heterogeneities. Therefore, we employ Z_{bio} for downstream analysis at the cellular level. In the exploration of condition-related genes, we employ Z_{cond}^{sp} for visualization and identification of associated genes. Z_{cond}^{sp} signifies condition-specific biological effects, highlighting heterogeneities between different conditions. As our focus lies on condition-specific genes, we use Z_{cond}^{sp} for downstream analysis at the gene level.

For the nine comparison methods, during downstream analysis of UMAP visualization, we use the recommended cell representation from each algorithm for plotting and clustering. Specifically, Seurat utilizes the integrated PCA latent layer. Harmony uses the integrated latent layer represented by ho.Z_corr. Scanorama uses the integrated latent layer denoted as integrated. DESC uses the recommended integrated latent layer X_umap0.8. scVI uses the integrated latent layer X_scVI. Cell BLAST uses the integrated latent layer X_latent. SCIDRL employs the integrated output latent layer represented by embed. scDisInFact uses the integrated cell latent layer denoted as z_cs. scINSIGHT uses the quantile-normalized module expression norm.W_2 as the integrated latent layer.

3.2 Detailed description of the real datasets

The Human Pancreas 2 (Type 2 Diabetes)[7] dataset is available on the European Bioinformatics Institute (EBI) platform with the accession number E-MTAB-5061. This dataset consists of pancreatic cells from male donors aged 22, 23, 25, 27, and 43, male donors aged 52 and 57 with type 2 diabetes, a female donor aged 48, and female donors aged 37 and 55 with type 2 diabetes. In total, the dataset contains 2,914 cells and 30,415 genes.

The Human Lung[8] dataset, accessible for download on the EBI platform under the accession number E-MTAB-130148, comprises lung cells from one 62-year-old patient

with Chronic Obstructive Pulmonary Disease (COPD), two 58-year-old healthy donors, and one 69-year-old healthy donor. In total, the dataset contains 3,566 cells and 19,206 genes.

The Human Pancreas[9] dataset is sourced from the GEO database and can be accessed using the accession numbers GSM2230757, GSM2230758, GSM2230759, and GSM2230760. It consists of pancreatic cells from a 17-year-old male donor, a 51-year-old female donor, a 38-year-old male donor, and a 59-year-old female donor with type 2 diabetes. In total, the dataset contains 8,569 cells and 20,125 genes. We utilized downsampling of the human pancreas to construct a specialized dataset with only one shared cell type. First, we selected samples from a normal individual and an individual with type 2 diabetes from the human pancreas, both sharing nine cell types: 'acinar', 'activated_stellate', 'delta', 'beta', 'ductal', 'alpha', 'endothelial', 'gamma', 'quiescent_stellate'. Second, we sampled each individual, removing the data of the last four cell types 'ductal', 'alpha', 'endothelial', 'gamma' for the normal sample and the first four cell types 'acinar', ' activated_stellate', 'delta', 'quiescent_stellate' for the diseased sample. Finally, we obtained a dataset named 'human pancreas-subset' containing only the shared 'beta' cell type. Each batch comprises four unique cell types, resulting in a total of 3125 cells.

The Mouse Mucosa[10] dataset, available on the EBI platform with the accession number E-CURD-52, contains nasal mucosa cells from two female mice stimulated with interferon-alpha and two male mice without stimulation. In total, the dataset contains 43,677 cells and 30,446 genes.

The Human Epithelium[11] dataset, accessible on the EBI platform under the accession number E-CURD-114, comprises airway epithelial cells from various donors. The donors include a 10-year-old non-smoking female, a 23-year-old mild-smoking female, a 35-year-old non-smoking female, a 57-year-old female heavy smoker, a 66-year-old heavy smoker female, a 68-year-old non-smoking female, a male child aged

22 with asthma exacerbation, a 27-year-old non-smoking male donor, a 38-year-old non-smoking male donor, a 44-year-old male heavy smoker, a 55-year-old male heavy smoker, a 59-year-old male heavy smoker, a 61-year-old male heavy smoker, a 64-year-old non-smoking male donor, and a 66-year-old non-smoking male donor. In total, the dataset contains 32,926 cells and 26,528 genes.

The Human Ductal[12, 13] dataset, originating from Project PRJCA001063, represents the transcriptome of human pancreatic ductal cells. It includes cells from 24 patients with primary pancreatic ductal adenocarcinoma (PDAC) and 11 healthy individuals. In total, the dataset contains 57,530 cells and 18,008 genes. The dataset can be downloaded from the following link: https://ngdc.cncb.ac.cn/biop-roject/browse/PRJCA001063.

3.3 Derivation of the loss function based on the multi-faced VAE

In order to compute the posterior distribution of the latent variable $\boldsymbol{Z}$, given the samples $\boldsymbol{X}$ and noise $\boldsymbol{B}$, where $\boldsymbol{Z}$ is composed of two components $\boldsymbol{Z}_{bio}$ and $\boldsymbol{Z}_{cond}$, representing the embedding variables in the shared biological latent space and the conditional latent space, respectively. For simplicity, let's denote index 1 as *bio* and index 2 as *cond*, such that $\boldsymbol{Z}_1 = \boldsymbol{Z}_{bio}$ and $\boldsymbol{Z}_2 = \boldsymbol{Z}_{cond}$. Assuming that the latent variable $\boldsymbol{Z}$ follows a prior distribution $p(\boldsymbol{Z})$ and a conditional distribution $p_\theta(\boldsymbol{X}|\boldsymbol{Z},\boldsymbol{B})$, we can use Bayes' theorem to obtain:

$$p(\boldsymbol{Z}|\boldsymbol{X},\boldsymbol{B}) = \frac{p_\theta(\boldsymbol{X}|\boldsymbol{Z},\boldsymbol{B})p(\boldsymbol{Z})}{p(\boldsymbol{X}|\boldsymbol{B})} = \frac{p_\theta(\boldsymbol{X}|\boldsymbol{Z},\boldsymbol{B})p(\boldsymbol{Z})}{\int p_\theta(\boldsymbol{X}|\boldsymbol{B},u)p(u)du}. \tag{6}$$

Directly computing $p(\boldsymbol{Z}|\boldsymbol{X},\boldsymbol{B})$ using equation 6 is challenging because the integral term in the denominator, $\int p_\theta(\boldsymbol{X}|\boldsymbol{B},u)p(u)du$ is difficult to calculate. Therefore,

variational inference is employed to estimate this distribution. We define a variational distribution $q_\phi(\boldsymbol{Z}|\boldsymbol{X}, \boldsymbol{B})$ to approximate the posterior distribution $p(\boldsymbol{Z}|\boldsymbol{X}, \boldsymbol{B})$. The Kullback-Leibler (KL) divergence is introduced to measure the similarity between these two distributions, where a smaller KL divergence indicates a closer match.

Combining prior knowledge with information extracted from the raw feature matrix $\boldsymbol{X}$, we typically assume that the conditional distribution $p_\theta(\boldsymbol{X}|\boldsymbol{Z}, \boldsymbol{B})$ follows a Gaussian distribution $\mathrm{N}(\mathrm{Dec}(\boldsymbol{Z}, \boldsymbol{B}), c\boldsymbol{I})$, where Dec is a deterministic function neural network defined by the parameters θ. Due to the intractable integral in the denominator, computing the posterior probability directly using Bayes' theorem is not feasible. Instead, we approximate the posterior distribution $p(\boldsymbol{Z}|\boldsymbol{X}, \boldsymbol{B})$ by using a variational distribution $q_\phi(\boldsymbol{Z}|\boldsymbol{X}, \boldsymbol{B})$. Assuming that the biological latent space and the conditional latent space can be separated, we can express the multi-faced variational distribution $q_\phi(\boldsymbol{Z}|\boldsymbol{X}, \boldsymbol{B})$[14]as:

$$q_\phi(\boldsymbol{Z}|\boldsymbol{X}, \boldsymbol{B}) = q_\phi(\boldsymbol{X}, \boldsymbol{B}) \prod_{j=1}^{J} q_\phi(\boldsymbol{Z}_j|\boldsymbol{X}, \boldsymbol{B}), \tag{7}$$

where J=2 and ϕ represents the parameters of the encoder.

To minimize the KL divergence $\mathrm{D}_{\mathrm{KL}}(q_\phi(\boldsymbol{Z}|\boldsymbol{X}, \boldsymbol{B}) \mid\mid p(\boldsymbol{Z}|\boldsymbol{X}, \boldsymbol{B}))$, we need to select an appropriate distribution from the family $\{q_\phi(\boldsymbol{Z}|\boldsymbol{X}, \boldsymbol{B})\}$. This leads to the following optimization problem:

$$\min_\phi \mathrm{D}_{\mathrm{KL}} \left(q_\phi(\boldsymbol{Z}|\boldsymbol{X}, \boldsymbol{B}) \mid\mid p(\boldsymbol{Z}|\boldsymbol{X}, \boldsymbol{B})\right). \tag{8}$$

Expanding the KL divergence:

$$\begin{aligned}
&\mathrm{D_{KL}}\left(q_\phi(\boldsymbol{Z}|\boldsymbol{X},\boldsymbol{B}) \;||\; p(\boldsymbol{Z}|\boldsymbol{X},\boldsymbol{B})\right) \\
&= \mathbb{E}_{z\sim q}[\log q_\phi(\boldsymbol{Z}|\boldsymbol{X},\boldsymbol{B})] - \mathbb{E}_{z\sim q}[\log p(\boldsymbol{Z}|\boldsymbol{X},\boldsymbol{B})] \\
&= \mathbb{E}_{z\sim q}[\log q_\phi(\boldsymbol{Z}|\boldsymbol{X},\boldsymbol{B})] - \mathbb{E}_{z\sim q}\left[\frac{\log p_\theta(\boldsymbol{Z}|\boldsymbol{X},\boldsymbol{B})p(\boldsymbol{Z})}{p(\boldsymbol{X}|\boldsymbol{B})}\right] \\
&= \mathbb{E}_{z\sim q}[\log q_\phi(\boldsymbol{Z}|\boldsymbol{X},\boldsymbol{B})] - \mathbb{E}_{z\sim q}[\log p(\boldsymbol{Z})] - \mathbb{E}_{z\sim q}[\log p_\theta(\boldsymbol{X}|\boldsymbol{Z},\boldsymbol{B})] \\
&\quad + \mathbb{E}_{z\sim q}[\log p(\boldsymbol{X}|\boldsymbol{B})] \\
&= \mathrm{D_{KL}}\left(q_\phi(\boldsymbol{Z}|\boldsymbol{X},\boldsymbol{B}) \;||\; p(\boldsymbol{Z})\right) - \mathbb{E}_{z\sim q}[\log p_\theta(\boldsymbol{X}|\boldsymbol{Z},\boldsymbol{B})] + \log p(\boldsymbol{X}|\boldsymbol{B}).
\end{aligned} \tag{9}$$

The term $\log p(\boldsymbol{X}|\boldsymbol{B})$ is a constant given the observed data. By rearranging the equation, we obtain:

$$\log p(\boldsymbol{X}|\boldsymbol{B}) - \mathrm{D_{KL}}\left(q_\phi(\boldsymbol{Z}|\boldsymbol{X},\boldsymbol{B}) \;||\; p(\boldsymbol{Z}|\boldsymbol{X},\boldsymbol{B})\right) = \mathbb{E}_{z\sim q}[\log p_\theta(\boldsymbol{X}|\boldsymbol{Z},\boldsymbol{B})] - \mathrm{D_{KL}}\left(q_\phi(\boldsymbol{Z}|\boldsymbol{X},\boldsymbol{B}) \;||\; p(\boldsymbol{Z})\right).$$

The right-hand side represents the evidence lower bound (ELBO), and minimizing the KL divergence on the left-hand side is equivalent to maximizing the ELBO[15]:

$$\begin{aligned}
&\min_\phi \mathrm{D_{KL}}\left(q_\phi(\boldsymbol{Z}|\boldsymbol{X},\boldsymbol{B}) \;||\; p(\boldsymbol{Z}|\boldsymbol{X},\boldsymbol{B})\right) \\
&= \max_{\phi,\theta} \mathrm{ELBO} \\
&= \max_{\phi,\theta} \Big\{\mathbb{E}_{z\sim q}[\log p_\theta(\boldsymbol{X}|\boldsymbol{Z},\boldsymbol{B})] - \mathrm{D_{KL}}\left(q_\phi(\boldsymbol{Z}|\boldsymbol{X},\boldsymbol{B}) \;||\; p(\boldsymbol{Z})\right)\Big\}.
\end{aligned} \tag{10}$$

For estimating $\mathbb{E}_{z\sim q}[\log p_\theta(\boldsymbol{X}|\boldsymbol{Z},\boldsymbol{B})]$, we can use Stochastic Gradient Variational Bayes (SGVB) estimation, which gives:

$$\mathbb{E}_{z\sim q}[\log p_\theta(\boldsymbol{X}|\boldsymbol{Z},\boldsymbol{B})] = \frac{1}{L}\sum_{l=1}^{J} \log p(\boldsymbol{X}|\boldsymbol{Z}^l,\boldsymbol{B}) = -\frac{||\boldsymbol{X}-\widetilde{\boldsymbol{X}}||}{2c} - k,$$

where L is the number of Monte Carlo samples in the SGVB estimation (we set L to 1), and k and c are constants.

Assuming $p(\boldsymbol{Z}_1)$ and $p(\boldsymbol{Z}_2)$ are standard Gaussian distributions, with $p(\boldsymbol{Z}_1) \sim \mathrm{N}(0, \boldsymbol{I}_{d_1\times d_1})$ and $p(\boldsymbol{Z}_2) \sim \mathrm{N}(0, \boldsymbol{I}_{d_2\times d_2})$. Similarly, $q_\phi(\boldsymbol{Z}_1|\boldsymbol{X},\boldsymbol{B})$ and $q_\phi(\boldsymbol{Z}_2|\boldsymbol{X},\boldsymbol{B})$ are Gaussian distributions, where $q_\phi(\boldsymbol{Z}_1|\boldsymbol{X},\boldsymbol{B}) \sim \mathrm{N}(\mu_1, \sigma_1^2)$ and $q_\phi(\boldsymbol{Z}_2|\boldsymbol{X},\boldsymbol{B}) \sim \mathrm{N}(\mu_2, \sigma_2^2)$. To compute the Kullback-Leibler divergence $\mathrm{D_{KL}}\left(q_\phi(\boldsymbol{Z}|\boldsymbol{X},\boldsymbol{B}) \,||\, p(\boldsymbol{Z})\right)$, we can adopt a Monte Carlo estimation:

$$
\begin{aligned}
&\mathrm{D_{KL}}\left(q_\phi(\boldsymbol{Z}|\boldsymbol{X},\boldsymbol{B}) \,||\, p(\boldsymbol{Z})\right)\\
&= \sum_{j=1}^{J}\Big[\mathrm{D_{KL}}\left(q_\phi(\boldsymbol{Z}_j|\boldsymbol{X},\boldsymbol{B}) \,||\, p(\boldsymbol{Z}_j)\right)\Big]\\
&= \mathrm{D_{KL}}\left(q_\phi(\boldsymbol{Z}_1|\boldsymbol{X},\boldsymbol{B}) \,||\, p(\boldsymbol{Z}_1)\right) + \mathrm{D_{KL}}\left(q_\phi(\boldsymbol{Z}_2|\boldsymbol{X},\boldsymbol{B}) \,||\, p(\boldsymbol{Z}_2)\right)\\
&= \mathbb{E}_{z_1\sim q}[\log q_\phi(\boldsymbol{Z}_1|\boldsymbol{X},\boldsymbol{B}) - \log p(\boldsymbol{Z}_1)] + \mathbb{E}_{z_2\sim q}[\log q_\phi(\boldsymbol{Z}_2|\boldsymbol{X},\boldsymbol{B}) - \log p(\boldsymbol{Z}_2)]\\
&= \mathbb{E}_{z_1\sim q}[\log q_\phi(\boldsymbol{Z}_1|\boldsymbol{X},\boldsymbol{B})] - \mathbb{E}_{z_1\sim q}[\log p(\boldsymbol{Z}_1)] + \mathbb{E}_{z_2\sim q}[\log q_\phi(\boldsymbol{Z}_1|\boldsymbol{X},\boldsymbol{B})] - \mathbb{E}_{z_2\sim q}[\log p(\boldsymbol{Z}_2)]\\
&= \int q_\phi(\boldsymbol{Z}_1|\boldsymbol{X},\boldsymbol{B})\log q_\phi(\boldsymbol{Z}_1|\boldsymbol{X},\boldsymbol{B})d\boldsymbol{Z}_1 - \int q_\phi(\boldsymbol{Z}_1|\boldsymbol{X},\boldsymbol{B})\log p(\boldsymbol{Z}_1)d\boldsymbol{Z}_1\\
&\quad + \int q_\phi(\boldsymbol{Z}_2|\boldsymbol{X},\boldsymbol{B})\log q_\phi(\boldsymbol{Z}_2|\boldsymbol{X},\boldsymbol{B})d\boldsymbol{Z}_2 - \int q_\phi(\boldsymbol{Z}_2|\boldsymbol{X},\boldsymbol{B})\log p(\boldsymbol{Z}_2)d\boldsymbol{Z}_2\\
&= \int \mathrm{N}(\mu_1,\sigma_1^2)\log \mathrm{N}(\mu_1,\sigma_1^2)d\boldsymbol{Z}_1 - \int \mathrm{N}(\mu_1,\sigma_1^2)\log \mathrm{N}(0,\boldsymbol{I}_{d_1\times d_1})d\boldsymbol{Z}_1\\
&\quad + \int \mathrm{N}(\mu_2,\sigma_2^2)\log \mathrm{N}(\mu_2,\sigma_2^2)d\boldsymbol{Z}_2 - \int \mathrm{N}(\mu_2,\sigma_2^2)\log \mathrm{N}(0,\boldsymbol{I}_{d_2\times d_2})d\boldsymbol{Z}_2\\
&= \Big[-\frac{I}{2}\log(2\pi) - \frac{1}{2}\sum_{i=1}^{d_1}\sum_{j=1}^{n}(1+\log(\sigma_1)_{ij}^2)\Big] - \Big[-\frac{I}{2}\log(2\pi) - \frac{1}{2}\sum_{i=1}^{d_1}\sum_{j=1}^{n}((\mu_1)_{ij}^2 + (\sigma_1)_{ij}^2)\Big]\\
&\quad + \Big[-\frac{I}{2}\log(2\pi) - \frac{1}{2}\sum_{i=1}^{d_2}\sum_{j=1}^{n}(1+\log(\sigma_2)_{ij}^2)\Big] - \Big[-\frac{I}{2}\log(2\pi) - \frac{1}{2}\sum_{i=1}^{d_2}\sum_{j=1}^{n}((\mu_2)_{ij}^2 + (\sigma_2)_{ij}^2)\Big]\\
&= \frac{1}{2}\sum_{i=1}^{d_1}\sum_{j=1}^{n}((\mu_1)_{ij}^2 + (\sigma_1)_{ij}^2 - 1 - \log(\sigma_1)_{ij}^2) + \frac{1}{2}\sum_{i=1}^{d_2}\sum_{j=1}^{n}((\mu_2)_{ij}^2 + (\sigma_2)_{ij}^2 - 1 - \log(\sigma_2)_{ij}^2)).
\end{aligned}
$$

Since our objective is to minimize the loss function, the target function can be expressed as:

$$
\begin{aligned}
loss_{\mathrm{VAE}} &= -\mathrm{ELBO} \\
&= \mathrm{D}_{\mathrm{KL}}\left(q_{\phi}(\boldsymbol{Z}|\boldsymbol{X},\boldsymbol{B}) \;||\; p(\boldsymbol{Z})\right) - \mathbb{E}_{z\sim q}[\log p_{\theta}(\boldsymbol{Z}|\boldsymbol{X},\boldsymbol{B})] \\
&= \sum_{i=1}^{m}\sum_{j=1}^{n} ||x_{ij} - \widetilde{x}_{ij}||^2 + \lambda\Big(\frac{1}{2}\sum_{i=1}^{d_1}\sum_{j=1}^{n}((\mu_1)_{ij}^2 + (\sigma_1)_{ij}^2 - 1 - \log(\sigma_1)_{ij}^2) \\
&\quad + \frac{1}{2}\sum_{i=1}^{d_2}\sum_{j=1}^{n}((\mu_2)_{ij}^2 + (\sigma_2)_{ij}^2 - 1 - \log(\sigma_2)_{ij}^2))\Big) \\
&\triangleq loss_1 + \lambda\ loss_2,
\end{aligned}
$$

where x_{ij} represents the element in the i-th row and j-th column of matrix $\boldsymbol{X}$; $\widetilde{x}_{ij}$ represents the element in the i-th row and j-th column of the reconstructed matrix $\widetilde{\boldsymbol{X}}$; the terms μ_{1ij} and σ_{1ij}^2 correspond to the i-th row and j-th column elements of matrix $\boldsymbol{\mu}_1$ and $\boldsymbol{\sigma}_1^2$, respectively, which represent the biological hidden Gaussian distribution; the terms μ_{2ij} and σ_{2ij}^2 correspond to the i-th row and j-th column elements of matrix $\boldsymbol{\mu}_2$ and $\boldsymbol{\sigma}_2^2$, respectively, representing the conditional hidden Gaussian distribution; λ denotes a hyperparameter.

References

[1] Carlson M, Falcon S, Pages H, Li N, et al. org. Hs. eg. db: Genome wide annotation for Human. R package version. 2019;3(2):3.

[2] Carlson M. org. Mm. eg. db: Genome wide annotation for Mouse. R package version 3.2. 3. Bioconductor London, United Kingdom: Genome Biology (BMC). 2019;.

[3] Hubert L, Arabie P. Comparing partitions. Journal of Classification. 1985;2:193–218. https://doi.org/https://doi.org/10.1007/BF01908075.

[4] Strehl A, Ghosh J. Cluster ensembles—a knowledge reuse framework for combining multiple partitions. Journal of Machine Learning Research. 2002;3(Dec):583–617. https://doi.org/https://search.ebscohost.com/login.aspx?direct=true&db=aph&AN=10257734&site=eds-live.

[5] Rousseeuw PJ. Silhouettes: a graphical aid to the interpretation and validation of cluster analysis. Journal of Computational and Applied Mathematics. 1987;20:53–65. https://doi.org/https://doi.org/10.1016/0377-0427(87)90125-7.

[6] Tran HTN, Ang KS, Chevrier M, Zhang X, Lee NYS, Goh M, et al. A benchmark of batch-effect correction methods for single-cell RNA sequencing data. Genome Biology. 2020;21(1):1–32. https://doi.org/https://doi.org/10.1186/s13059-019-1850-9.

[7] Segerstolpe Å, Palasantza A, Eliasson P, Andersson EM, Andréasson AC, Sun X, et al. Single-cell transcriptome profiling of human pancreatic islets in health and type 2 diabetes. Cell Metabolism. 2016;24(4):593–607. https://doi.org/https://doi.org/10.1016/j.cmet.2016.08.020.

[8] Vieira Braga FA, Kar G, Berg M, Carpaij OA, Polanski K, Simon LM, et al. A cellular census of human lungs identifies novel cell states in health and in asthma. Nature Medicine. 2019;25(7):1153–1163. https://doi.org/https://doi.org/10.1038/s41591-019-0468-5.

[9] Baron M, Veres A, Wolock SL, Faust AL, Gaujoux R, Vetere A, et al. A single-cell transcriptomic map of the human and mouse pancreas reveals inter-and intra-cell population structure. Cell Systems. 2016;3(4):346–360. https://doi.org/https://doi.org/10.1016/j.cels.2016.08.011.

[10] Ziegler CG, Allon SJ, Nyquist SK, Mbano IM, Miao VN, Tzouanas CN, et al. SARS-CoV-2 receptor ACE2 is an interferon-stimulated gene in human airway epithelial cells and is detected in specific cell subsets across tissues. Cell. 2020;181(5):1016–1035. https://doi.org/https://doi.org/10.1016/j.cell.2020.04.035.

[11] Goldfarbmuren KC, Jackson ND, Sajuthi SP, Dyjack N, Li KS, Rios CL, et al. Dissecting the cellular specificity of smoking effects and reconstructing lineages in the human airway epithelium. Nature Communications. 2020;11(1):2485. https://doi.org/https://doi.org/10.1038/s41467-020-16239-z.

[12] Peng J, Sun BF, Chen CY, Zhou JY, Chen YS, Chen H, et al. Single-cell RNA-seq highlights intra-tumoral heterogeneity and malignant progression in pancreatic ductal adenocarcinoma. Cell Research. 2019;29(9):725–738. https://doi.org/https://doi.org/10.1038/s41422-019-0195-y.

[13] Yang K, Yang T, Yu J, Li F, Zhao X. Integrated transcriptional analysis reveals macrophage heterogeneity and macrophage-tumor cell interactions in the progression of pancreatic ductal adenocarcinoma. BMC Cancer. 2023;23(1):199. https://doi.org/https://doi.org/10.1186/s12885-023-10675-y.

[14] Falck F, Zhang H, Willetts M, Nicholson G, Yau C, Holmes CC. Multi-facet clustering variational autoencoders. Advances in Neural Information Processing Systems. 2021;34:8676–8690. https://doi.org/https://doi.org/10.48550/arXiv.2106.05241.

[15] Maddison CJ, Mnih A, Teh YW. The concrete distribution: A continuous relaxation of discrete random variables. arXiv:161100712. 2016;https://doi.org/https://doi.org/10.48550/arXiv.1611.00712.
